# Unraveling the impact of SARS-CoV-2 mutations on immunity: insights from innate immune recognition to antibody and T cell responses

**DOI:** 10.3389/fimmu.2024.1412873

**Published:** 2024-12-10

**Authors:** Rafael Bayarri-Olmos, Adrian Sutta, Anne Rosbjerg, Mie Mandal Mortensen, Charlotte Helgstrand, Per Franklin Nielsen, Laura Pérez-Alós, Beatriz González-García, Laust Bruun Johnsen, Finn Matthiesen, Thomas Egebjerg, Cecilie Bo Hansen, Alessandro Sette, Alba Grifoni, Ricardo da Silva Antunes, Peter Garred

**Affiliations:** ^1^ Laboratory of Molecular Medicine, Department of Clinical Immunology, Copenhagen University Hospital, Rigshospitalet, Copenhagen, Denmark; ^2^ Recombinant Protein and Antibody Unit, Copenhagen University Hospital, Rigshospitalet, Copenhagen, Denmark; ^3^ Recombinant Technologies, Novo Nordisk A/S, Måløv, Denmark; ^4^ Center for Vaccine Innovation, La Jolla Institute for Immunology, La Jolla, CA, United States; ^5^ Department of Medicine, Division of Infectious Diseases and Global Public Health, University of California, San Diego (UCSD), La Jolla, CA, United States; ^6^ Department of Clinical Medicine, Faculty of Health and Medical Sciences, University of Copenhagen, Copenhagen, Denmark

**Keywords:** SARS-CoV-2, variants of concern, delta, omicron, mannose-binding lectin, MBL, immune imprinting, vaccine

## Abstract

Throughout the COVID-19 pandemic, the emergence of new viral variants has challenged public health efforts, often evading antibody responses generated by infections and vaccinations. This immune escape has led to waves of breakthrough infections, raising questions about the efficacy and durability of immune protection. Here we focus on the impact of SARS-CoV-2 Delta and Omicron spike mutations on ACE-2 receptor binding, protein stability, and immune response evasion. Delta and Omicron variants had 3–5 times higher binding affinities to ACE-2 than the ancestral strain (KD_wt_ = 23.4 nM, KD_Delta_ = 8.08 nM, KD_BA.1_ = 4.77 nM, KD_BA.2_ = 4.47 nM). The pattern recognition molecule mannose-binding lectin (MBL) has been shown to recognize the spike protein. Here we found that MBL binding remained largely unchanged across the variants, even after introducing mutations at single glycan sites. Although MBL binding decreased post-vaccination, it increased by 2.6-fold upon IgG depletion, suggesting a compensatory or redundant role in immune recognition. Notably, we identified two glycan sites (N717 and N801) as potentially essential for the structural integrity of the spike protein. We also evaluated the antibody and T cell responses. Neutralization by serum immunoglobulins was predominantly mediated by IgG rather than IgA and was markedly impaired against the Delta (5.8-fold decrease) and Omicron variants BA.1 (17.4-fold) and BA.2 (14.2-fold). T cell responses, initially conserved, waned rapidly within 3 months post-Omicron infection. Our data suggests that immune imprinting may have hindered antibody and T cell responses toward the variants. Overall, despite decreased antibody neutralization, MBL recognition and T cell responses were generally unaffected by the variants. These findings extend our understanding of the complex interplay between viral adaptation and immune response, underscoring the importance of considering MBL interactions, immune imprinting, and viral evolution dynamics in developing new vaccine and treatment strategies.

## Introduction

Almost two years after the official end of the COVID-19 pandemic, the emergence of novel SARS-CoV-2 variants with enhanced viral fitness has not been stopped. 1These novel variants, classified by the WHO as variants of interest (VOI) or, in the worst cases, variants of concern (VOC), are characterized by a persistent accumulation of mutations primarily in their spike proteins. By mediating the binding to the angiotensin-converting enzyme 2 (ACE-2) receptor in host cells, the spike protein is the main determinant of infection (1). Furthermore, the spike protein is the antigen used in most available vaccines and the main target for protective neutralizing antibodies (nAbs), and as such, mutations occurring within the spike gene may give rise to variants with enhanced transmissibility and immune evasive capabilities (2). Indeed, the variability of the spike protein is probably to blame for the rise of breakthrough infections that have prolonged the COVID-19 pandemic.

The B.1.617.2 strain was identified in India in the state of Maharashtra in October 2020 (3, 4), rapidly spreading through India and worldwide, outcompeting the alpha VOC. It was designated as the Delta VOC by WHO in May 2021 (5). The Delta spike protein carried mutations never seen before in previous VOCs, and known mutations affecting sites important for antibody-mediated neutralization (6–8). In the period from July to December 2021, it accounted for virtually all new COVID-19 cases, only to be swiftly replaced by the B.1.1.529 strain. This strain was first identified in South Africa at the end of November 2021. Despite border closures and travel restrictions, B.1.1.529 spread unhindered worldwide and was designated as the Omicron VOC in a matter of days (9). Early reports showed that Omicron had gained a fitness advantage over Delta in terms of enhanced transmissibility (10, 11), but resulted in milder infections and fewer hospitalizations (12). This decline in virulence may be the result of attenuated replication of Omicron in the upper and lower respiratory tracts compared to the ancestral Wuhan strain—hereafter referred to as wild-type (wt)—and the Delta variant due to its lower fusogenicity (13, 14). This first Omicron wave was caused by the BA.1 subvariant (Pango lineage B.1.1.529.1), which was itself almost immediately replaced by the BA.2 subvariant (Pango lineage B.1.1.529.2). Worryingly, timely reports monitoring the transmission of these subvariants showed not only that BA.2 was substantially more transmissible than BA.1 (15–17), but that it also caused more severe infections (16).

While BA.1 and BA.2 are classified as Omicron sublineages and not independent variants, they have marked differences. The BA.1 subvariant harbors more than 30 mutations on its spike protein (i.e. four times the number carried by Delta). Of these, 15 map to the receptor binding domain (RBD), the mediator of the interaction with the human ACE-2 receptor in host cells (1), and the main target for nAbs (18, 19). Some residues, such as K417, E484, and N501, were already mutated in the previous VOCs Alpha (B.1.1.7), Beta (B.1.351), and Gamma (P.1), and have been found to potently diminish neutralization by convalescent and vaccinee sera (20, 21). The BA.2 subvariant has 28 substitutions and 1 deletion in the spike protein, 21 of these shared with BA.1, one with Delta (T19I), and seven unique (Δ24–26, A27S, V213G, S371F, F376A, D405N, R408S).

Adding to the concerning succession of evolving SARS-CoV-2 variants, in 2022 it was reported three recombinant lineages—XD, XE, XF—probably originating from the co-infection in a single host of the Delta, BA.1, and BA.2 variants (22). The XD recombinant lineage, first identified in December 2021, is a Delta (AY.4) genome with the Omicron BA.1 spike sequence and has been reported in Belgium, the Netherlands, Denmark, and France. The recombinant lineage XF is also a Delta and BA.1 recombinant, with a breakpoint at the non-structural protein 3 (NSP3). The XE lineage is a recombinant of BA.1 and BA.2, containing BA.1 mutations in NSP1 to 6, and BA.2 mutations in the rest of the genome. XF and XE are mostly associated with UK-sequenced samples and there is no evidence of transmission in other EU/EEA countries.

The dramatic accumulation of mutations seen in the Delta and Omicron spikes has raised concerns that protection from antibodies or T cells generated from previous infections or vaccination may be severely compromised. Here, we aimed to provide a comprehensive overview of the significance of the spike mutations of the Delta, BA.1, and BA.2 variants, focusing on ACE-2 affinity, protein stability, and glycosylation; and their contribution to evasion of recognition by vaccine- and infection-induced antibodies, T cells, and the humoral innate immune pattern recognition molecule mannose-binding lectin (MBL). MBL is one of the main activators of the complement cascade of the innate immune system (23), recently shown to bind to the SARS-CoV-2 spike protein and mediate complement activation and direct opsonization (24). This interaction is highly dependent on the glycan shield of the spike protein. Beyond the masking of individual epitopes, changes in the glycan shield may lead to widespread alterations in the antigenic surface that extend far from the specific glycan attachment sites (25). To identify the potential MBL binding sites on the SARS-CoV-2 spike protein, we selected 12 experimentally verified N-glycan sites and performed site-directed mutagenesis to evaluate their potential for escaping MBL recognition. Additionally, we evaluated the impact of immune imprinting on antibody-mediated neutralization and T cell recognition of the Delta and Omicron variants.

## Results

### Emergence of VOCs and defining mutations

**Figure 1:**
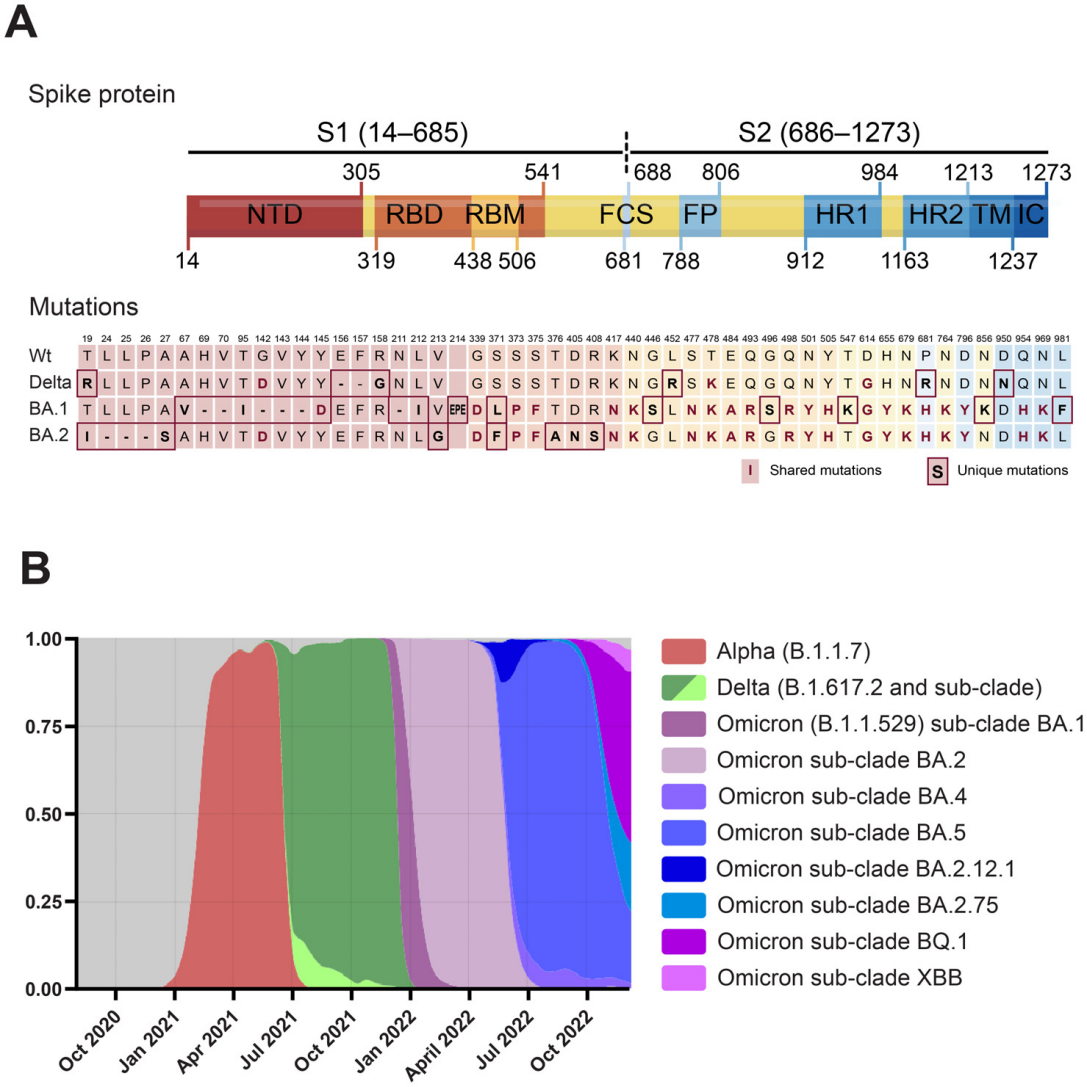
**(A)** Domain structure of the SARS-CoV-2 spike protein and location of the Delta, Omicron BA.1, and Omicron BA.2-defining mutations. Numbering and domain boundaries according to the spike wt. NTD, N-terminal domain; RBD, receptor binding domain; RBM, receptor binding motif; FCS, furin cleavage site; FP, fusion peptide; HR1/2, heptad repeats 1/2; TM, transmembrane domain; IC, intracellular domain. **(B)** Frequency distribution of sequences from SARS-CoV-2 VOCs in Denmark from October 2020 to 2^nd^ of January 2023. Modified from https://covariants.org. Only VOCs with frequencies above 0.05 are plotted. Data for panels A and B from the Nextstrain GISAID database (https://nextstrain.org/ncov/gisaid/global) (157, 158).

The location of all consensus mutations in the spike protein of VOCs Delta (B.1.617.2), and Omicron BA.1 (B.1.1.529.1) and BA.2 (B.1.1.529.2) are depicted in **Figure 1A**. Delta contains eight mutations in the spike protein: the three novel substitutions T19R, G142D, and R158G, and the also novel deletion 157–158del, clustering in the N-terminal domain (NTD) “supersite” recognized by all known anti-NTD nAbs (26, 27); L452R, and T478K in the RBD, both shown to impair antibody binding (6–8); and D614G, P681R, and D950N, which may improve viral fitness beyond antibody evasion (28–30). The Omicron BA.1 spike was at the time, the most mutated spike, accumulating 34 mutations. Of these, seven are in the NTD, such as the 69–70del which results in S-gene target failure in the TaqPath RT-PCR and allowed to identify both variants (31); the 142–144del and Y145D mutations mapping to the N3 loop of the NTD antigenic supersite (26), and a novel EPE insertion, not observed previously in any SARS-CoV-2 lineage, in what has been identified as a recurrent insertion region (32). Ten out of the 15 RBD mutations cluster in the receptor binding motif (RBM), forming the ACE-2 binding interface (33), such as the affinity-enhancing N501Y and E484A, likely highly immune evasive as the E484K present in previous VOC (20, 21). Moreover, most of these mutations are found in exposed, antibody-accessible regions of the spike trimer, and will probably confer antibody resistance. The mutations N679K and P681H, in the vicinity of the furin cleavage site, and shared with the BA.2 sublineage, have been shown to compromise the proteolytic cleavage of the S1/S2 subunits required for membrane fusion and host cell infection (34). Including the N679K and P681H, the Omicron BA.2 spike shares 20 mutations with BA.1. It also harbors seven additional mutations compared to BA.1: three substitutions and the 24–26del within the NTD, and four mutations in the RBD (but outside the RBM).

These mutations and others mapping outside the spike—and beyond the scope of this work, have given the Delta and Omicron VOCs the upper hand in the arms race that has characterized the COVID-19 pandemic, with novel variants that have been succeeding one another at increasing frequencies (**Figure 1B**). As in other countries, in the period between July 2021 to December 2021, the vast majority of SARS-CoV-2 sequences in Denmark were identified as Delta. During December, Omicron BA.1 cases increased exponentially and then declined in mid-January to be overtaken by the BA.2 sublineage.

### Impact of mutations in ACE-2 interaction and RBD stability

**Figure 2:**
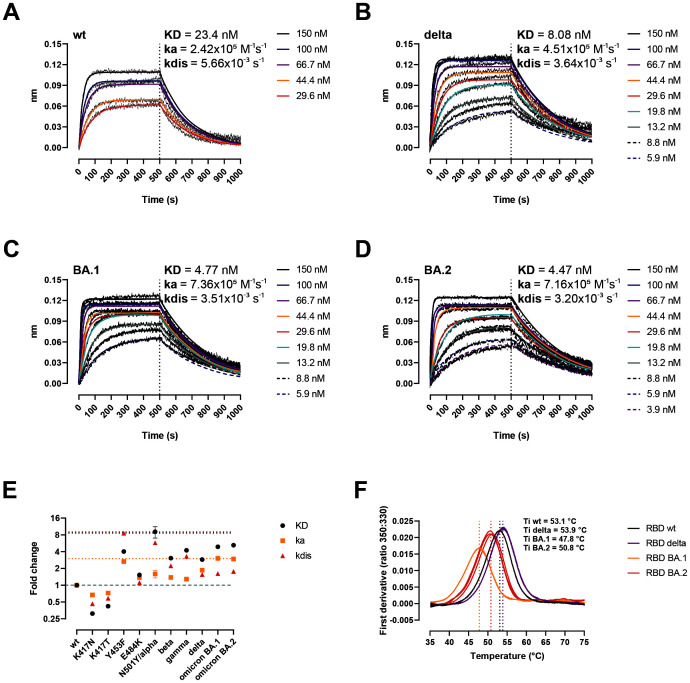
Biochemical impact of RBD mutations in Delta, BA.1, and BA.2 variants. **(A–D)** BLI sensorgrams of RBD wt **(A)**, Delta **(B)**, BA.1 **(C)**, and BA.2 **(D)**. ACE-2-Fc was immobilized on anti-human Fc capture sensors. ACE-2-immobilized sensors were dipped into serial dilutions of RBD (association 500 s), followed by only buffer (dissociation 500 s). **(E)** Kinetic parameters of single mutations and VOCs determined by BLI here and somewhere else (20, 21, 38). Data represent fold-change compared to the wt. Horizontal dotted black, yellow, and red lines signal the top KD, ka, and kdis values. Horizontal dashed grey line signals the baseline (no change compared to the wt). **(F)** Thermal denaturation curves of the RBD wt, Delta, BA.1, and BA.2 variants. Data are represented as individual first derivative curves of the 350:330 nm ratio from three repeats. Vertical dotted lines represent the inflection temperatures (Ti).

The binding affinity of the spike protein towards the human ACE-2 receptor is a determinant of SARS-CoV-2 infectivity (35–37). Therefore, we analyzed the binding kinetics of the RBD variants to ACE-2 using biolayer interferometry (BLI) (**Figure 2**). Compared to the wt, the RBD Delta displayed approx. a 4-fold higher affinity (KD_wt_ = 23.4 nM, KD_Delta_ = 8.08 nM) (**Figures 2A, B**), and the Omicron BA.1 and BA.2 variants a 5-fold higher affinity than the wt (KD_BA.1_ = 4.77 nM, KD_BA.2_ = 4.47 nM) (**Figures 2C, D**). All three RBD variants had similar dissociation rates (kdis), ranging from 3.2x10^−3^ s^−1^ for BA.2 to 3.64x10^−3^ s^−1^ for Delta, representing a 1.76- to 1.55-fold improvement compared to the wt. Comparing the kinetic parameters of all VOCs identified to date (20, 21), as well as the RBD mutation Y453F identified in mink (38), the RBD alpha remains the VOC with the highest binding affinity (approx. 8-fold improvement over the wt), followed by BA.2 (5.2-fold) and BA.1 (4.9-fold) (**Figure 2E**). The Omicron variants however, emerged as the fastest ACE-2 binders to date (i.e. highest binding rate, ka) (ka_BA.1_ = 7.36x10^5^ M^−1^s^−1^, ka_BA.2_ = 7.16x10^5^ M^−1^s^−1^). Protein stability has been identified as another key determinant of infectivity (39). We evaluated the RBD stability by nanoDSF by monitoring the intrinsic fluorescence ratio at 350 and 330 nm (**Figure 2F**). The RBD Delta was more stable than the wt (Ti = 53.9 vs 53.1°C, respectively), while the BA.1 and BA.2 had inflection temperatures that were 5.3 and 2.3°C lower than that of the wt strain. Thus, the two RBD-mapping mutations in the Delta have a net positive effect in both ACE-2 affinity and stability, while the BA.1 and BA.2 mutations have opposite effects in ACE-2 interaction and RBD stability.

### Glycan analyses of the spike protein of SARS-CoV-2 wt, Delta, and omicron

**Figure 3:**
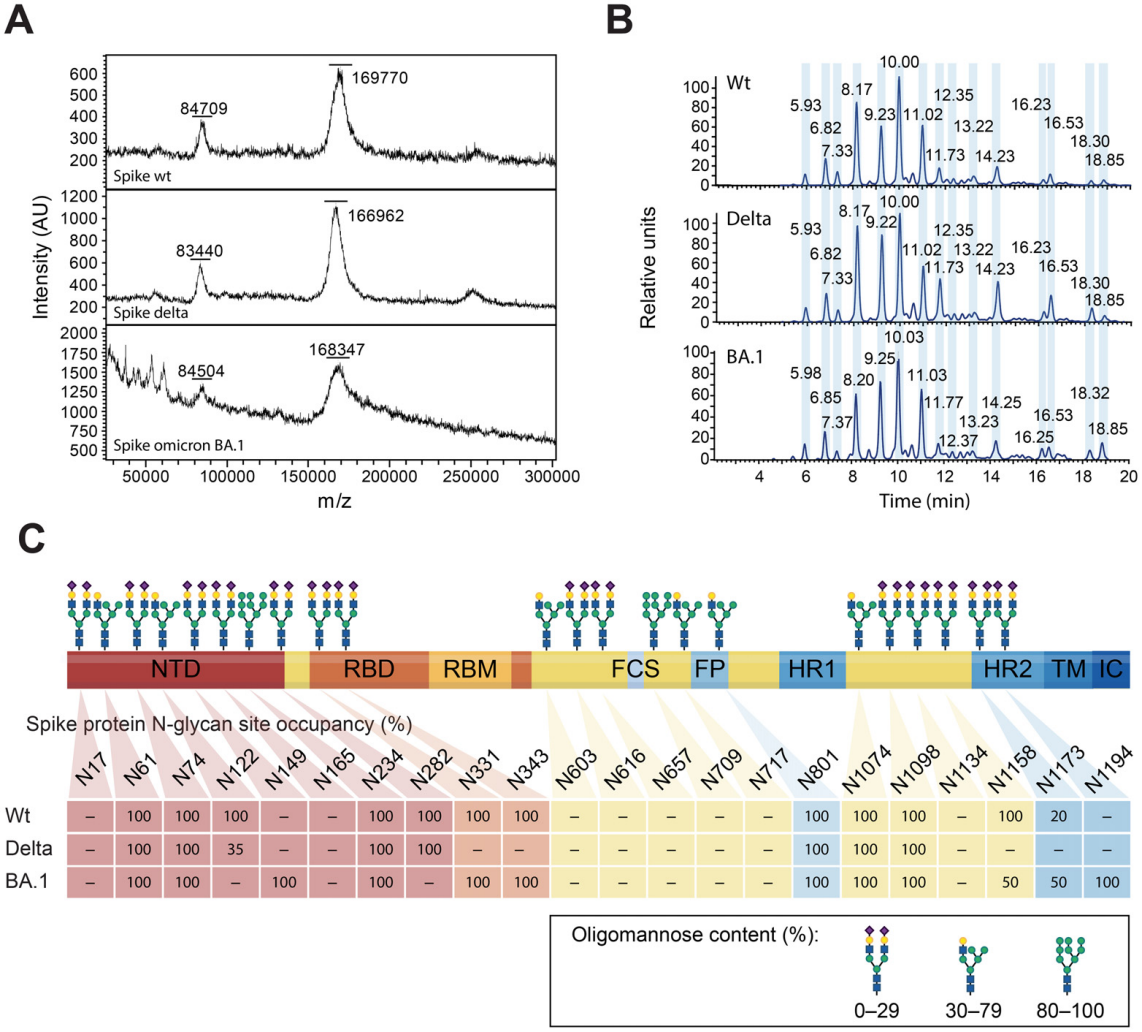
MS-based glycan analyses of the glycan shield of the spike wt, Delta, and Omicron BA.1. **(A)** MW determination by MALDI-MS. The two mayor peaks represent the intact spike peptide chain and probably the processed S1 subunit. **(B)** Released N-glycan profile after PNGaseF treatment and fluorescent labelling. The magnitude of the peaks is represented as relative units. **(C)** N-glycan site-specific occupancy, as determined by peptide mapping, for the spike wt, Delta, and Omicron BA.1. Positions that were not resolved are noted as “–”. N-glycans are represented as complex (oligomannose content ≤ 29%), hybrid (30–79%), and oligomannose (≥ 80%), from Watanabe et al., (40).

The spike protein, the most polymorphic of the SARS-CoV-2 structural proteins, is heavily glycosylated (40). It has been proposed that it is via its N-glycan sites that the humoral innate recognition molecule MBL binds to and neutralizes SARS-CoV-2 (24). Thus, recombinant spike wt, Delta, and BA.1 were analyzed by MS to determine whether mutations within the spike protein may alter its glycan shield, impairing MBL recognition (**Figure 3**). Intact mass analyses by direct MALDI-MS revealed a molecular weight (MW) of 167–169.8 kDa for wt, Delta, and BA.1 produced in ExpiCHO cells (**Figure 3A**). The 30.5–33.1 kDa difference with the predicted aa-derived mass represents total glycosylation, which means that no major differences in the extent of glycosylation were observed between the three variants. Similarly, LC-MS analyses of the N-glycan release profile after PNGaseF digestion show a very similar peak profile for all three proteins (**Figure 3B**). O-glycosylation, analyzed on fully reduced and de-glycosylated proteins using PNGaseF, was below the detection limit. Finally, we compared the site-specific glycosylation of the spike variants by LC-MS after trypsin digestion (**Figure 3C**). Even though not all sites were covered by the trypsin-based peptide map, we observed no significant difference with respect to site-specific glycan profiles between the three variants.

### Recognition of SARS-CoV-2 spike variants by the humoral innate immune molecule MBL

**Figure 4:**
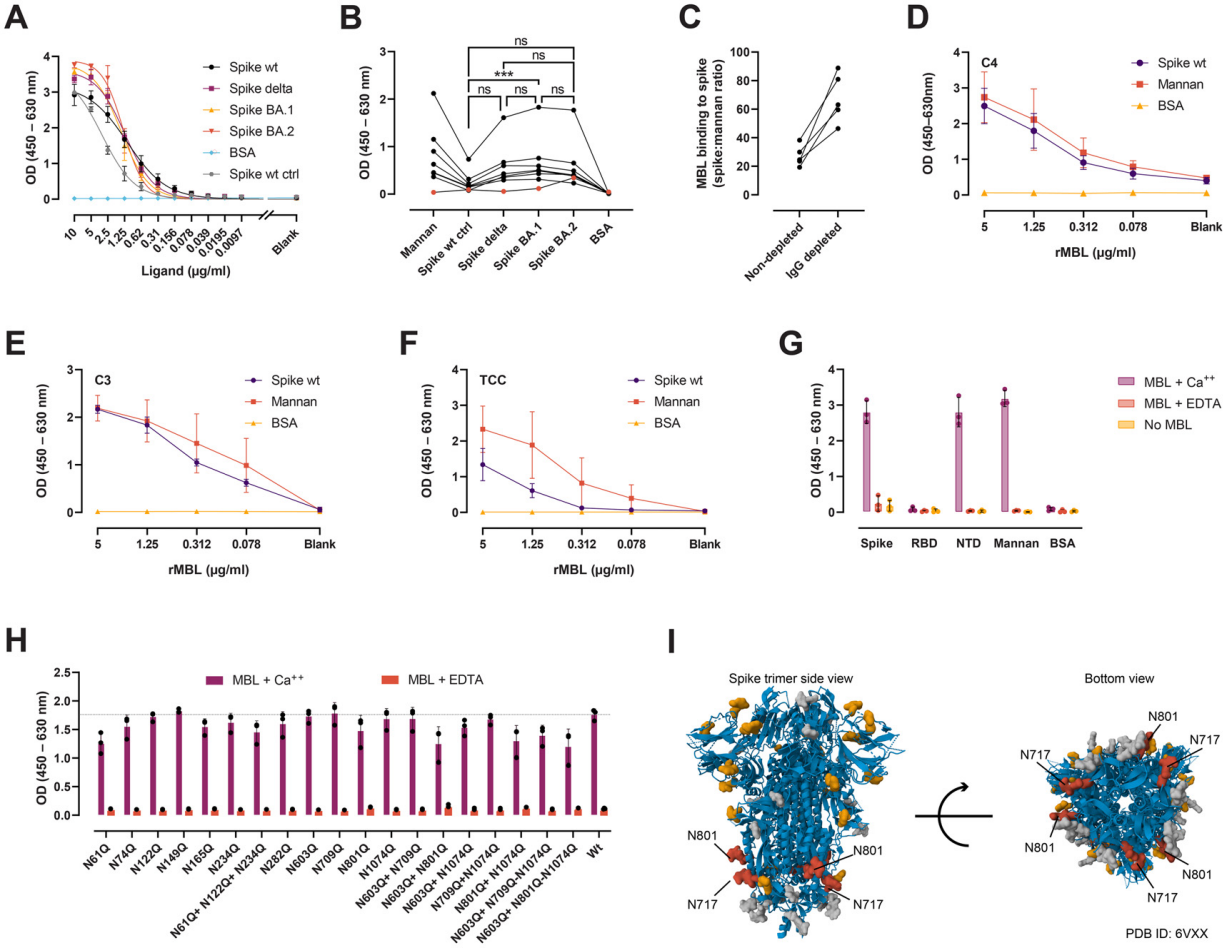
MBL interaction with spike. **(A)** Detection of rMBL bound to coated, 2-fold dilutions of spike wt, Delta (both *in-house*), BA.1, BA.2 (both from AcroBiosystems). BSA was used as negative control, and spike wt from NIBSC as positive control (ctrl). **(B)**. Binding of native MBL from naïve sera from seven healthy, MBL-sufficient individuals (black dots). Naïve, MBL-defect serum was used as negative control (red dots). Friedman test with Dunn’s multiple comparisons. ***, *p* < 0.001. **(C)** Binding of native MBL from vaccinee plasma before and after total IgG depletion (*n* = 5). Data is normalized to mannan binding to correct for MBL loss after running the plasma through protein G agarose columns. **(D–F)** MBL-dependent complement deposition on spike. Serial dilutions of rMBL were applied to coated spike, mannan, or BSA, followed by naïve MBL-defect serum as a source of complement. Complement activation was measured as C4 **(D)**, C3 **(E)**, and TCC **(F)** deposition. **(G)** Calcium-dependent interaction between MBL and full-length spike, spike NTD, and spike RBD. EDTA was used to chelate calcium. No MBL was used as negative control. Mannan and BSA were used as positive and negative ligand controls, respectively **(A–G)**. **(H)** MBL binding to spike N-glycan mutants from ExpiCHO supernatants captured with an anti-spike mAb (*in-house*) under calcium sufficient (MBL + Ca^++^) or deficient (MBL + EDTA) conditions. Kruskal-Wallis test with Dunn’s multiple comparisons. **(I)** Structural representation of the spike protein (PDB ID: 6VXX (159)) with N-glycans (molecular surface) shown in grey. Highlighted in yellow are those positions evaluated in **(G)** by site-directed mutagenesis, and in red, those that impaired protein production and stability. Data from the Protein Data Bank (RCSB PDB) (https://www.rcsb.org/) (160). Created with Mol* Viewer (161). ns, not significant.

MBL is a recognition molecule of the complement system, capable of binding to carbohydrates on pathogens and other surfaces, and driving the generation of activated fragments of C4, C3, and C5, and the assembly of the terminal complement complex (TCC) via the lectin pathway (23). MBL has been shown to bind to the SARS-CoV-2 spike protein and mediate complement activation and direct opsonization. Here we evaluated whether the Delta and Omicron BA.1 and BA.2 variants escape from MBL recognition and map the critical N-glycan sites for MBL binding. Detection of rMBL bound to serial dilutions of spike wt, Delta, BA.1, and BA.2 showed specific and comparable binding curves (**Figure 4A**). Similarly, native MBL from naïve sera was found to interact with all three variants to the same extent (**Figure 4B**), except for a significant difference between spike BA.1 and the spike wt control. Of note, binding of native MBL to spike after vaccination was greatly reduced and increased 2.6-fold (range 1.89–3.27, *n* = 5) after total IgG depletion (**Figure 4C**). Binding of rMBL to spike, followed by naïve MBL-defect serum—to ensure that complement activation occurs exclusively via MBL—resulted in C4 (**Figure 4D**), C3 (**Figure 4E**), and TCC (**Figure 4F**) deposition. Finally, we sought to define the region of the spike protein recognized by the lectin activity of MBL. MBL was found to bind to the full-length spike and the NTD in a calcium-dependent fashion, but not to the RBD (**Figure 4G**). We performed site-directed mutagenesis to systematically remove selected N-glycan sites and evaluated the binding to the single, double, and triple N-glycan-deficient mutants (**Figure 4H**). Of the 12 positions evaluated as single, double, and triple mutants, none was critical for MBL recognition. However, removal of N717 and N801 resulted in much decreased recombinant protein yields, impaired thermal stability, and protein degradation (**Supplementary Figure 1**; **Figure 4I**).

### Evolution of antibody titers and antibody avidity in vaccinees and infected individuals

**Figure 5:**
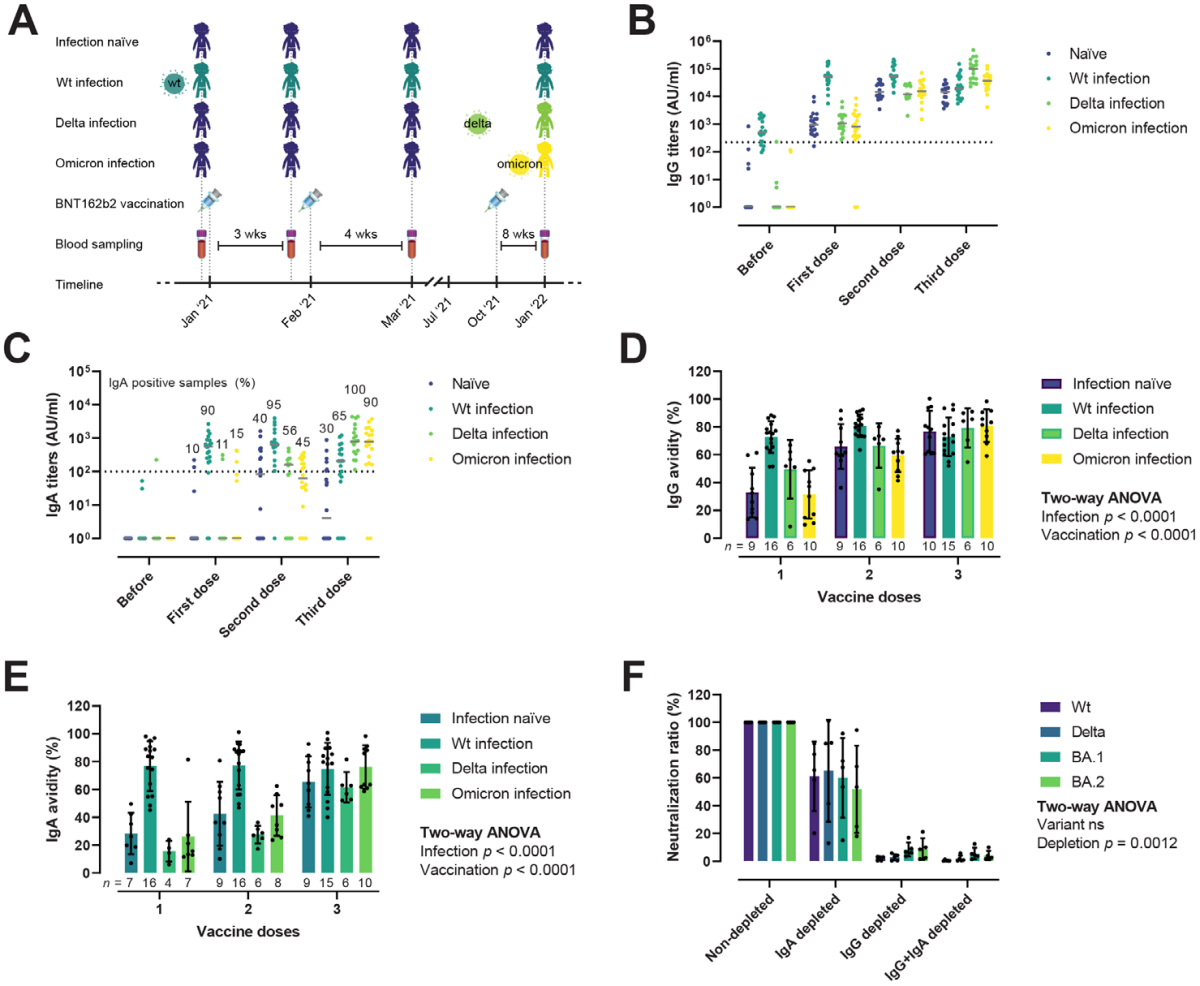
IgG and IgA responses after infection and vaccination. **(A)** Overview of the vaccinee cohort. Donors were grouped in infection-naïve (*n* = 20), wt infection (*n* = 20), Delta infection (*n* = 18), and Omicron infection (*n* = 20). Blood samples were collected before vaccination, after the first, second, and third doses of the BNT162b2 vaccine. **(B, C)** Evolution of IgG **(B)** and IgA **(C)** RBD-specific titers, reported as arbitrary units (AU)/ml, after infection and vaccination. Horizontal solid lines represent median. Horizontal dotted lines represent the threshold for positivity. **(D, E)** Avidity maturation of IgG **(D)** and IgA **(E)**. Ordinary Two-way ANOVA. Data is represented as mean ± SD. **(F)** IgG and IgA nAbs contribution to the neutralization potency of hybrid immune sera (*n* = 5), plotted as the ratio (in percentage) of IgG, IgA, and IgG+IgA depleted to non-depleted sera. Two-way ANOVA with the Geisser-Greenhouse correction. Data is represented as mean ± SD.

We monitored RBD-specific IgG and IgA titers and avidity in longitudinal samples from individuals with different infection histories to study how infection and vaccination shapes the antibody responses (**Table 1**). Samples were collected before BNT162b2 vaccination, three weeks after the first dose (median 22 days), one month after the second dose (median 31 days), and two months after the third dose/booster (median 83 days). We found no statistically significant differences between groups with respect to sex, age, or BMI. When comparing days between vaccination and the last blood drawn, individuals who got infected with Omicron received their first, second, and third doses later than the other groups. Individuals were classified as infection-naïve, or having had a wt/Delta/Omicron infection according to the date of their positive RT-PCR test and the presence of anti-protein N antibodies (**Figure 5A**). As expected, IgG titers increased after successive vaccine and booster doses (**Figure 5B**). Two months after the third dose, antibody levels had plateaued both in those that had experienced a prior infection before the vaccine, and in infection-naïve individuals. Those with Delta or Omicron infections showed enhanced IgG responses after the third dose, probably boosted by the recent infection. IgA responses were also boosted gradually following successive vaccine doses (**Figure 5C**). After the first dose, only 10–15% of the infection-naïve samples (which at this time point includes naïve, Delta and Omicron infection groups) had IgA responses above the threshold of positivity. One month after the second dose, these numbers rose to 40–56%, but decreased slightly in the infection-naïve samples two months after the third dose (30%). In contrast, 90% of the hybrid immune group with a prior infection (wt) developed an IgA response already after one vaccine dose, which was maintained one month after the second dose (95%) and decreased slightly two months after the third dose (65%). Infection with Delta or Omicron after a complete two-dose vaccination was very effective at boosting IgA responses, with 100% and 90% IgA positive in the Delta and Omicron hybrid immune groups respectively, compared to 30% in infection-naïve.

**Table 1:** Demographic data and characteristics of the study cohort at the last collection round.

	Total	Infection-naive	WT infection	Delta infection	Omicron infection	P-value
	(*n* = 78)	(*n* = 20)	(*n* = 20)	(*n* = 18)	(*n* = 20)	
Sex						
Female	69 (88.5%)	18 (90.0%)	19 (95.0%)	15 (83.3%)	17 (85.0%)	0.6633 ^a^
Male	9 (11.5%)	2 (10.0%)	1 (5.0%)	3 (16.7%)	3 (15.0%)	
Age (years)						
Median (IQR)	49.0 (39.3–59.0)	57.0 (44.5–63.3)	51.5 (44.8–57.3)	45.5 (37.5–53.3)	46.0 (39.0–53.8)	0.162 ^a^
<40	22 (28.2%)	3 (15.0%)	4 (20.0%)	7 (38.9%)	8 (40.0%)	0.2524 ^b^
>40-55	29 (37.2%)	6 (30.0%)	9 (45.0%)	7 (38.9%)	7 (35.0%)	
>55	27 (34.6%)	11 (55.0%)	7 (35.0%)	4 (22.2%)	5 (25.0%)	
BMI						
Median (IQR)	24.2 (21.9–26.9)	23.4 (22.0–26.1)	25.0 (22.8–28.4)	23.3 (21.9–24.9)	25.8 (22.7–26.9)	0.4186 ^a^
Underweight	2 (2.6%)	0 (0%)	0 (0%)	1 (5.6%)	1 (5.0%)	0.1874 ^b^
Normal	38 (48.7%)	11 (55.0%)	9 (45.0%)	11 (61.1%)	7 (35.0%)	
Overweight	19 (24.4%)	4 (20.0%)	5 (25.0%)	2 (11.1%)	8 (40.0%)	
Obese	6 (7.7%)	0 (0%)	4 (20.0%)	1 (5.6%)	1 (5.0%)	
Missing	13 (16.7%)	5 (25.0%)	2 (10.0%)	3 (16.7%)	3 (15.0%)	
Time between first vaccine dose and last blood drawn (days)						
Median (IQR)	379 (370–390)	378 (371–384)	378 (370–383)	378 (369–389)	391 (384–398)	0.005519 ^a^
Time between second vaccine dose and last blood drawn (days)						
Median (IQR)	349 (342–360)	348 (340–352)	347 (342–350)	348 (339–356)	361 (352–367)	0.003691 ^a^
Time between third vaccine dose and last blood drawn (days)						
Median (IQR)	83.0 (75.0–91.0)	83.5 (78.8–91.5)	81.0 (73.8–88.0)	51.0 (41.0–76.0)	92.5 (89.0–97.0)	<1e-04 ^a^
Missing	1 (1.3%)	0 (0%)	0 (0%)	1 (5.6%)	0 (0%)	
Time between positive PCR and last blood drawn (days)						
Median (IQR)	90.5 (19.0–628)	N.A.	649 (628–662)	90.5 (82.5–109)	17.0 (15.0–19.0)	<1e-04 ^c^
Time between first and second vaccine dose (days)						
Median (IQR)	30.0 (29.0–32.0)	31.0 (30.0–32.0)	30.0 (28.0–31.0)	31.0 (30.0–33.0)	30.5 (29.8–32.0)	0.2711 ^a^
Time between second and third vaccine dose (days)						
Median (IQR)	267 (259–276)	261 (257–269)	269 (264–272)	297 (267–309)	267 (257–272)	0.0004014 ^a^
Missing	1 (1.3%)	0 (0%)	0 (0%)	1 (5.6%)	0 (0%)	

IQR Interquartile range.

N.A. Not Applicable.

a Kruskal–Wallis test (two-sided) between infection-naive participants, participants infected before Omicron, participants infected with Delta, and participants infected with Omicron.

b Chi-squared test (two-sided) between infection-naive participants, participants infected before Omicron, participants infected with Delta, and participants infected with Omicron.

c Kruskal–Wallis test (two-sided) between participants infected before Omicron, participants infected with Delta, and participants infected with Omicron.

We next evaluated the avidity maturation of RBD-specific IgG and IgA antibodies in a subset of vaccinees that developed both IgG and IgA responses (**Figures 5D, E**). As shown for antibody titers, avidity was significantly enhanced by both vaccination and infection, albeit the influence of each differed for IgG (**Figure 5D**) and IgA (**Figure 5E**). Vaccination and infection had comparable effects in terms of IgG avidity, e.g., two vaccine doses were equivalent to one dose and a previous infection. Regarding IgA, infection appeared to be the main driver of the avidity maturation, with the hybrid immune group presenting qualitatively better IgA responses already after the first vaccine dose compared to infection-naïve after one or two vaccine doses. After the third dose, we observed no statistically significant differences in the avidity of IgG and IgA between infection-naïve and hybrid immune individuals.

To investigate the relative contribution of IgG and IgA to the viral neutralization activity of immune sera, we selectively depleted IgG, IgA, or both by passing sera from five vaccinees with a recent Omicron infection through protein G or peptide M columns, and measured neutralization of the RBD wt, Delta, BA.1, and BA.2 (**Figure 5F**). A reduction/inhibition of the interaction between RBD or spike and ACE-2 measured in ELISA was used as a proxy for nAbs (41). After IgA depletion, neutralization potency was reduced to 57.2–65% of the non-depleted sera. In contrast, IgG or IgG+IgA double depleted sera retained only a minor neutralizing activity (3.3–11.2% and 0.5–6.7%, respectively). There was no significant difference in the relative contribution of IgG and IgA to the neutralization of the different variants. In fact, even though all donors had an Omicron infection, removal of IgA resulted in comparable drops in neutralization of the wt/Delta and Omicron variants, suggesting that IgA may target conserved regions of the RBD shared between the vaccine and the strain that caused the infection.

Taken together, these results indicate that hybrid immunity (i.e., immunity generated by infection and vaccination) induce quantitatively and qualitatively superior antibody responses, and that serum viral neutralization in fully vaccinated individuals is mostly driven by IgG.

### Neutralization by sera from naïve and convalescent vaccinees

**Figure 6:**
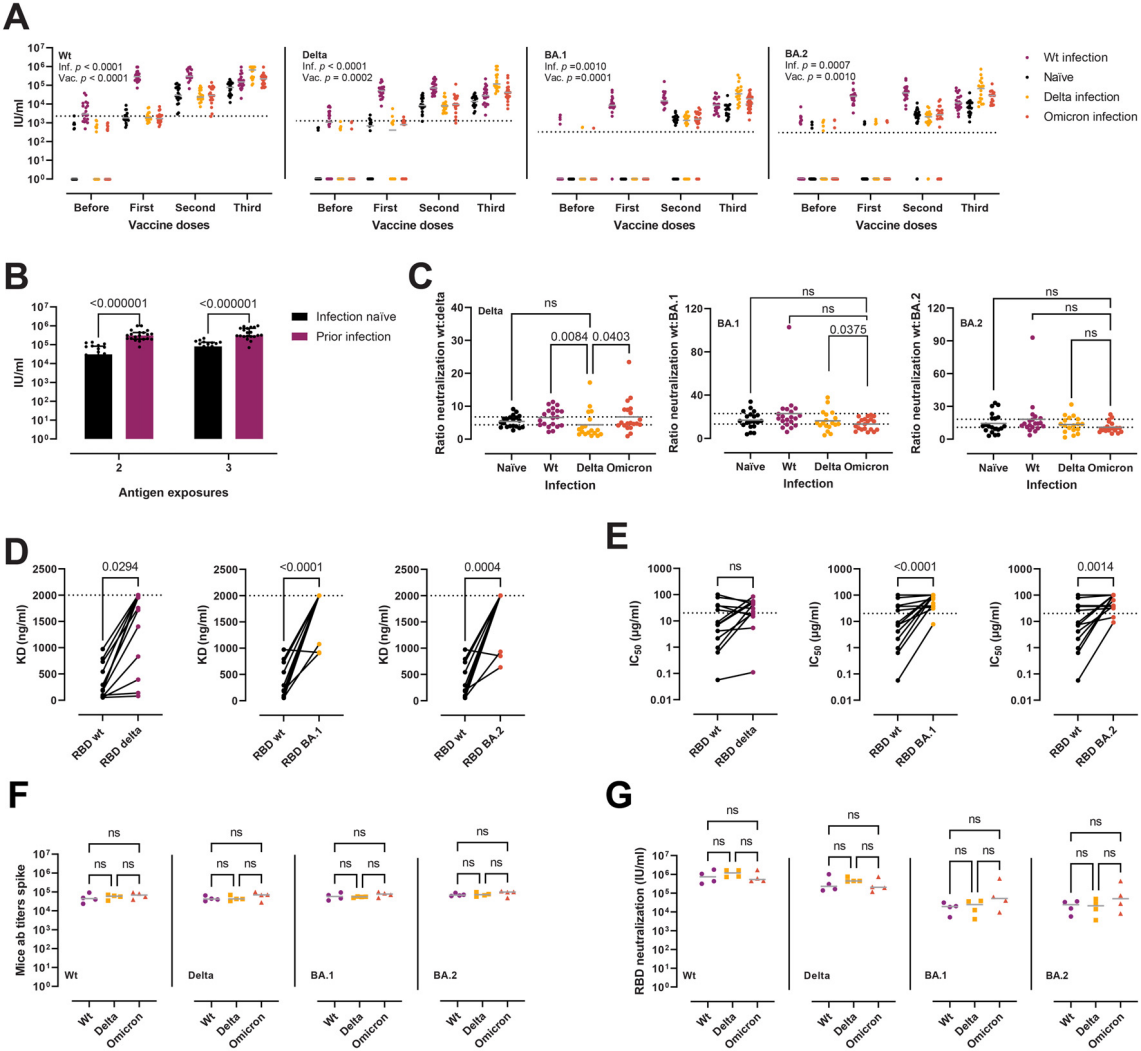
Neutralization of RBD wt, Delta, BA.1, and BA.2 after vaccination and infection. **(A)** Neutralization potency of sera, reported as IU/ml, from infection-naïve individuals (*n* = 20), wt infection (*n* = 20), Delta (*n* = 18), and Omicron (*n* = 20), against RBD wt, Delta, BA.1, and BA.2. Two-way ANOVA with the Geisser-Greenhouse correction. Horizontal grey lines represent the median. Horizontal dotted lines represent the threshold for positivity. **(B)** Comparison between the neutralization potency of sera against RBD wt from infection-naïve individuals (*n* = 20) and sera from those with a previous infection (*n* = 20) from panel **(A)** Two antigen exposures refer to complete vaccination or infection + first dose. Three exposures refer to complete vaccination + booster or complete vaccination + infection. Mann-Whitney tests, with a two-stage linear step-up procedure of Benjamini, Krieger, and Yekutieli. Data are presented as median with interquartile range. **(C)** Antibody evasion gains of the Delta and Omicron variants plotted as the ratio of the neutralization of RBD wt to RBD Delta, BA.1, and BA.2 from panel **(A)** Kruskal-Wallis with Dunn’s multiple corrections. Horizontal grey lines represent the mean. Dashed lines indicate the highest and lowest mean values. Outliers identified by ROUT with Q = 1% (G [*n* = 7], H [*n* = 3]). **(D, E)** Affinity **(D)** and neutralization potency **(E)** of a panel of murine mAbs (*n* = 14) towards RBD wt, Delta, BA.1, and BA.2. Horizontal dotted lines indicate the maximum concentration of mAbs used in the assays. Friedman tests with Dunn’s multiple comparisons. **(F, G)** Antibody titers **(F)** and neutralization potency of sera **(G)** in a murine model of heterologous prime-boost vaccination. Mice were divided into wt (three doses of spike wt, *n* = 4), Delta (two doses of wt followed by a boost with spike Delta, *n* = 4), and Omicron (two doses of wt followed by a boost with spike Omicron, *n* = 4). Kruskal-Wallis with Dunn’s multiple corrections. ns, not significant.

After assessing the possibility of escape from humoral innate immune recognition, we questioned whether the Delta and Omicron variants present enhanced immune evasion of nAb responses, and if so, to which extent. For all variant RBDs, the neutralizing potency of sera increased, as expected, after each vaccine dose (**Figure 6A**). However, it appeared to reach a plateau after two doses, with no major gains with the third dose/booster in infection-naïve individuals. Likewise, in those individuals with a prior infection, we did not observe any improvement in neutralization after the first vaccine dose (i.e. two exposures). The same pattern was conserved across all three RBD variants, with a slight neutralization reduction against Delta and a more dramatic loss against BA.1 and BA.2. After the first dose, 46%, 100%, and 81% of the infection-naïve samples had no detectable nAbs against Delta, BA.1, and BA.2, respectively. After the complete two-dose vaccination, 1.7%, 27.6% and 10.3% of the samples remained negative. The neutralization potency of sera against the RBD and the full-length spikes correlated strongly for all variants, as well as the neutralization potency and RBD-specific IgG and IgA titers (**Supplementary Figure 2**). Moreover, the potency of nAbs induced by hybrid immunity remained superior at each dose, even when correcting for antigen exposures (**Figure 6B**).

To quantify the immune evasion properties of the variants, and to evaluate whether infection after vaccination induces variant specific antibody responses, we calculated the fold change in neutralization of the RBD Delta and Omicron variants compared to the wt in individuals with a complete vaccination plus booster and different infection histories (**Figure 6C**). The Delta variant displayed a mean decrease in neutralization of 5.8-fold compared to the wt (95% confidence interval [CI] = 5–6.6). The Omicron BA.1 was the most effective at avoiding neutralization, with a mean 17.4-fold reduction (95% CI = 14.6–20.2), while the Omicron BA.2 presented a mean 14.2-fold reduction (11.6–16.8), placing it between Delta and BA.1 in terms of antibody evasion. Delta infected experienced a lower decrease of delta-specific neutralization when compared to wt (*p* = 0.0084) or omicron infection (p = 0.0403), but not when compared to non-infected, while omicron infection provided no gains in variant-specific neutralization, which is suggestive of immune imprinting. Next, we assessed the affinity and neutralization capacity of a previously reported panel of 14 murine mAbs raised after immunization with spike or RBD from the ancestral strain (41) (**Figures 6D, E**). The Delta variant avoided recognition by 3/14 (21%) mAbs and impaired the binding of other 7/14 (50%) (**Figure 6D**). Four mAbs (29%) remained unaffected. On the other hand, the BA.1 and BA.2 Omicron variants avoided recognition/severely hindered the binding of most of the antibodies (12/14, 86%). Only two mAbs remained unperturbed by the BA.1 and BA.2 mutations. Similar results were obtained when evaluating the escape from neutralization (**Figure 6E**). From the eight mAbs with IC_50_ against the RBD wt below 20 µg/ml (the highest amount used in the assay), six displayed a marked reduction in neutralization potency and two were unaffected. Of the rest of the mAbs with a more modest neutralizing activity (IC_50_ > 20 µg/ml), 5/14 (36%) retained their activity towards Delta. A more dramatic escape from neutralization was recorded for the Omicron variants, with only one and two mAbs retaining activities below 20 µg/ml towards BA.1 and BA.2, respectively.

Finally, in order to scrutinize the effect of imprinting in antibody responses, we evaluated antibody titers and nAbs in a murine model of heterologous prime-boost vaccination, where mice where immunized thrice with the spike wt, or twice and boosted with either the Delta or Omicron spikes. We observed no significant differences in anti-spike wt/Delta/BA.1/BA.2 antibody titers (**Figure 6F**), nor in nAb responses against the variants (**Figure 6G**).

### Evaluation of T cell responses to SARS-CoV-2 wt and VOCs

**Figure 7:**
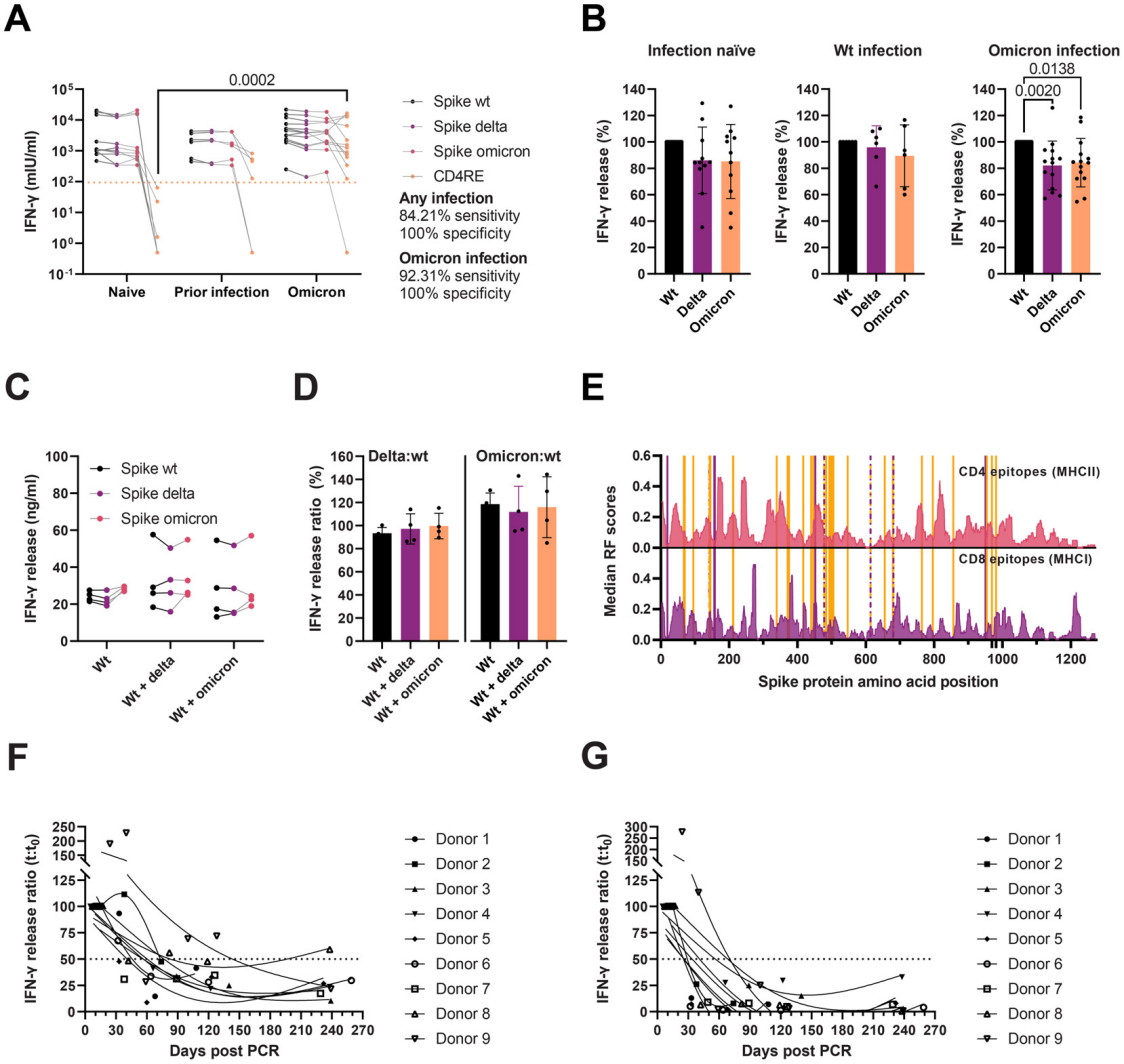
Cellular immunity to SARS-CoV-2 wt, Delta, and Omicron. **(A)** IFN-γ release after whole blood stimulation from infection-naïve (*n* = 11), individuals with a wt infection (*n* = 6) or a recent Omicron infection (*n* = 14), with peptide MPs covering the wt, Delta, and Omicron spike proteins, and CD4 T cell peptides restricted to the remainder of the proteome (CD4RE). Multiple Kruskal-Wallis tests with Dunn’s multiple comparisons corrections. Dotted line represents the threshold for positivity. **(B)** Ratio (%) of T cell responses after whole blood stimulation with spike peptide pools from panel A in infection-naïve, individuals with a wt infection, or an Omicron infection. Friedman tests with Dunn’s multiple comparisons. **(C)** IFN-γ release from splenocyte cultures from mice immunized thrice with spike wt (Wt) (*n* = 4), twice with spike wt followed by spike Delta (Delta) (*n* = 4), or twice with spike wt followed by spike Omicron BA.1 (Omicron) (*n* = 4). Two-way ANOVA with the Geisser-Greenhouse correction. **(D)** Variant-specific T cell responses from panel C plotted as the ratio of IFN-γ release after spike Delta MP stimulation to spike wt (left) or as the spike Omicron MP stimulation to spike wt (right). Kruskal-Wallis with Dunn’s multiple comparisons. Only statistically significant differences are plotted **(A–D)**. **(E)** Median response frequency (RF) scores for CD4 and CD8 epitopes from the SARS-CoV-2 spike protein. Data from the Immunome Browser (www.iedb.org) (45). Delta and Omicron mutated residues are marked in purple and orange, respectively. **(F, G)** Waning of T cell responses against spike wt **(F)** or CD4RE **(G)** peptide pools monitored for eight months after an Omicron infection (*n* = 9). Solid lines represent 3-knot smoothing splines. Horizontal dotted lines indicate the 50% response.

To estimate the impact of the Delta and Omicron spike mutations on T cell immunity, we stimulated whole blood using peptide MPs [described elsewhere (42–44)] covering different regions of the SARS-CoV-2 proteome from individuals with a complete vaccination who were either: 1) infection-naïve, 2) SARS-CoV-2 wt infected before the first vaccine dose, or 3) Omicron infected after the second vaccine dose (breakthrough infection). Briefly, we used four different MPs: three MPs of overlapping peptides covering the entire wt, Delta, and Omicron BA.1 spike proteins; and an experimentally-defined MP from the remainder of the proteome (CD4RE) specific for CD4 T cells. Whole blood stimulation using spike MPs elicited potent T cell responses, measured as released IFN-γ (mlU/ml), in all vaccinees (**Figure 7A**). Stimulation using the CD4RE MP comprising peptides outside the spike, and thus outside the vaccine antigen, allowed us to discriminate any previous infection with 84.21% sensitivity and 100% specificity, or a more recent Omicron infection with 92.31% sensitivity and 100% specificity. By comparing the T cell responses against the spike MPs from the Delta and Omicron spikes to the wt (**Figure 7B**), we observed a mild reduction irrespective of infection history—namely, in infection-naïve, responses against the Delta and Omicron were 86% (95% CI = 69.1–103) and 85.2% (66.3–104); in those with a prior infection, 95.8% (76.6–113) and 89.5% (64.8–114); and in recently infected with Omicron, 82.1% (71.4–92.8) and 84.3% (73.7–94.9). Of note, we observed no significant difference in T cell reactivity against the Delta and Omicron spike MP in those with an Omicron infection, nor in T cell reactivity against the Omicron spike in the different donor groups, suggesting that immune imprinting from vaccination may had dampened the induction of Omicron-specific T cells after infection. We investigated this further in the murine heterologous prime-boost vaccination model described previously. To capture antigen-specific memory and effector T cell responses, spleens were collected two weeks after the booster dose, and stimulated overnight with spike MPs (**Figures 7C, D**). As seen with vaccinees, mice receiving a Delta or Omicron boost did not present enhanced variant-specific T cell responses, measured as released IFN-γ.

We also characterized the secreted cytokine profile of activated T cells after spike wt, Delta, and Omicron (CD4 and CD8 T cells) and CD4RE peptide stimulation (CD4 T cells) (**Supplementary Figure 3**). The levels of 27 cytokines were measured by Luminex and compared to the levels of IFN-γ determined by ELISA as proxy for T cell activation. The levels of IFN-γ correlated strongly with the levels of IFN-γ (*ρ* = 0.8855) measured by Luminex, IL-2 (*ρ* = 0.8829), and IP-10 (*ρ* = 0.8544). The levels of these three cytokines after CD4RE peptide pool stimulation in vaccinated individuals non-infected (*n* = 8) or recovered from a recent Omicron infection (*n* = 11), could discriminate infection with 100% specificity and sensitivities ranging from 72.73% to 84.82%. A third of the cytokines (9/27) were found at concentrations below or close to the lower quantification limit (i.e. IL-5, VEGF, GM-CSF, IL-7, IL-10, IL-12, IL-13, IL-15, IL-17), while the rest (15/27) were expressed constitutively in full blood.

Response frequency (RF) scores from the Immune Epitope Database’s (IEDB) Immunome Browser Tool, (www.iebd.org) (45), can be used to identify immunodominant regions within a given antigen. To evaluate the putative impact of the Delta and Omicron BA.1 spike mutations on T cell recognition and activation, we overlapped the mutated residues on the position-specific RF scores for CD4 and CD8 epitopes in the spike antigen (**Figure 7E**). CD8 presents a slightly more widespread recognition pattern than CD4, which has distinct immunodominant regions (such as 166–180, 235–249, 313–320, 346–355, and 813–826, RF ≥ 0.3). The Delta L452R mutation occurs in a relatively immunodominant MHCI and MHCII epitope region (448–456 and 451–465, respectively), which may impair recognition by both CD8 and CD4 T cells. The Omicron BA.1 spike, on the other hand, harbors several mutations in the vicinity of MHCI (S375F) and MHCII (G339D, G446S, N764K) immunodominant regions, but none directly overlapping.

Finally, we evaluated the durability of T cell responses after Omicron infection in a group of fully vaccinated individuals. Samples were collected one to two weeks after recovery, and then again one, two, four, and eight months afterwards. Whole blood was stimulated with spike (**Figure 7F**) or CD4RE (**Figure 7G**) peptide pools. T cell responses appeared to wane rapidly within the first three months, with a half-life of around 40–80 days in most convalescent vaccinees (8/9). A single donor, with high T cell activity 40 days after the first positive RT-PCR test, displayed proportionately prolonged responses. While cell activation after both spike and CD4RE peptide stimulation decreased with time, responses against the latter decayed more rapidly (mean half-life spike = 81.87 days [95% CI 18.54–145.2], mean half-life CD4RE = 20.12 days [95% CI 5.46–34.78], *p* = 0.0078), and reached a lower baseline by month four than spike responses (8.2% vs 39.5% of the magnitude of the acute response for CD4RE and spike stimulation, respectively).

## Discussion

Several times throughout the COVID-19 pandemic, new viral variants have emerged with a remarkable ability to escape antibody responses (infection-induced, vaccine-induced, and/or antibody-based prophylactics), resulting in periodic waves of breakthrough infections (46). This has led to the question of whether the immune protection generated by the original vaccines targeting the ancestral/wt strain is a suitable match for the rapid evolution of the virus, or whether variant-specific vaccines should be used instead. However, the efficacy of variant-specific vaccines is conditioned by how cross-reactive the immune response generated by such vaccines would be when facing yet another variant, and by the extent of humoral and cellular immune imprinting. Studies of vaccinated and unvaccinated individuals infected with Omicron BA.1 reveal that Omicron-induced antibodies are poorly cross-reactive against other VOC (16), with this loss of reactivity ameliorated by prior vaccination (47). So far, data from Pfizer–BioNTech and Moderna bivalent vaccines, which target the ancestral and BA.1 strains, have been regarded as “underwhelming”, with neutralizing titers against the BA.1 merely 1.5–1.75 fold higher than those generated by the monovalent vaccine (48). When evaluated against the more recent Omicron variants, such as BA.2, BA.4, BA.5, and BA.2.75, bivalent vaccines resulted in no significant improvement in nAb titers (49, 50). Taken together, these findings suggest that deployment of Omicron-based vaccines, or other highly divergent SARS-CoV-2 strains, in immune-naïve individuals may induce poorly cross-reactive antibody responses, while Omicron boosters in vaccinees may be of limited use due to the imprinted responses from the ancestral strain-based vaccines. In this respect, our variant-specific neutralization results revealed that individuals with an Omicron breakthrough infection were not better at neutralizing BA.1/BA.2. Similarly, a recent Omicron infection did not appear to boost cell responses against the Omicron spike, and donors who have had an Omicron breakthrough infection presented a comparable reduction in T cell activation (15.7%) as infection-naïve (14.8%) after whole blood stimulation with a BA.1 spike-specific peptide pool. This decrease in the magnitude of T cell responses is in line with other reports (42, 51–53). Finally, we modelled a Delta and Omicron exposure after vaccination in mice and evaluated their antibody and cellular responses. Mice that encountered the Delta or Omicron spike antigen after two doses of the ancestral spike had comparable antibody titers and nAb responses against the corresponding variant, as well as T cell responses after splenocyte stimulation using Delta or Omicron peptide pools. We propose that the reason behind the failure to develop Delta and Omicron-specific responses and the limited efficacy of bivalent boosters, is one and the same: immune imprinting from the ancestral strain. Imprinting is being acknowledged by a growing body of literature (48–50, 54–59). Acute antibody responses after an Omicron breakthrough infection are biased towards the ancestral strain and manifest high levels of somatic hypermutation, indicating activation of pre-existing vaccine-induced memory B-cells, and a limited induction of *de novo* Omicron-specific B-cell responses (55–60). Antibody responses remain dominated by public clones also at later time points, as evidenced by a report on the evolution of the antibody response up to six months after an Omicron BA.1 breakthrough infection (58).

Here we have also studied the effect of Delta and Omicron BA.1 and BA.2 mutations in the kinetics of the RBD/ACE-2 interaction. BLI analyses revealed KDs of 8.08 nM for Delta, 4.77 nM for BA.1, and 4.47 nM for BA.2, all superior to the ancestral strain. Our binding affinity results, wherein BA.1 and BA.2 RBD mutations result in an increased ACE-2 affinity compared to the original strain, as well as the slightly lower affinity of BA.1 compared to BA.2 (61), are in agreement with other recent works (62–64). However, other authors have reported a slight loss of binding affinity for Omicron compared to Delta (65, 66). By also comparing with our previous binding kinetics data from the alpha, beta, and gamma RBDs, we noted that ACE-2 affinity peaked with the alpha variant at a point in the pandemic where the immune status of the population was for the most part naïve. Subsequent VOCs have accumulated further mutations in the RBD, such as the K417N/T in the beta and gamma variants, trading ACE-2 affinity for antibody escape (21, 67). The fact that the spike protein accumulates 43% and 61% of the Delta and Omicron-defining mutations when it accounts for only 13% of the SARS-CoV-2 proteome, and that some of these mutations have emerged independently in different VOCs and in patients with chronic infections (68–71), is suggestive of adaptive (convergent) evolution (69). In the case of Omicron, most of the spike-mapping mutations have been shown by deep mutational scanning to impair antibody neutralization (72, 73). In a population where pre-existing immunity is widespread, antibody escape may be of greater importance to viral transmissibility than receptor affinity. This trade-off has become quite prevalent in the highly mutated BA.1, where most mutations have either a neutral (G339D, S371L, S373P, S375F, N440K, T478K, E484A) or a deleterious effect (K417N, G446S, Q493R, G496S, and Y505H) on ACE-2 binding affinity, but are compensated by epistatic interactions with a few affinity-enhancing mutations (S477N < Q498R < N501Y) (i.e. deleterious mutations tend to become neutral in the presence of other mutations) (63). Moreover, we report a marked decrease in the thermal stability of the Omicron BA.1 RBD compared to their wt and Delta counterparts, and increased degradation of recombinant BA.1 spike (as evidenced in our MALDI-MS plot), and BA.1 RBD (data not shown). The introduction of basic residues in the BA.1 RBD makes it more susceptible to degradation by proteolytic enzymes, such as trypsin and chymotrypsin (74). Considering that stabilizing mutations, such as the prevalent D614G, promote infectivity (75), spike stability and its consequences on SARS-CoV-2 infectivity and virulence, may yet be another opportunity cost resulting from improvements in antibody resistance. Of note, we report improved ka—but not kdis—for the Omicron variants compared to all previous VOCs, and we hypothesize that fast binding rates may be more important for effective cell infection than longer receptor occupancy times. Recent reports have shown that while Omicron replicates less efficiently in the lungs than Delta and the wt strain (13, 14), it presents an enhanced replication in bronchi and nasal epithelium (76, 77). Thus, the higher density of Omicron viral particles in the upper respiratory tract, from where they can be readily shed, together with its marked gains in immune evasion, are likely the key factors that have led to the swift dominance of Omicron over all other VOCs.

Monitoring of glycosylation of emerging viral variants may be relevant for the identification of evolutionary patterns of viral immune evasion. Host glycosylation of viral proteins is important for protein folding, stability, viral tropism, and antigenicity (78). Changes in glycosylation are informative of alterations in protein structure (due to steric hindrances of glycan-processing enzymes), which may in turn compromise conformational antibody epitopes. Similarly, the addition or removal of glycan sites may lead to epitope masking (79, 80). Here, we asked whether the Delta and Omicron BA.1 and BA.2 spikes carried changes in their glycan shields that may impact innate humoral recognition by the pattern recognition molecule MBL of the complement system. We found little variability in the glycan shields of the wt, Delta, and Omicron BA.1 spikes. While some groups have reported an association between alleles resulting in lower MBL levels and a more severe clinical course of COVID-19 infection (81–84), disagreements remain in the literature and the relevance of MBL in COVID-19 progression remains disputed (24, 85–89). Here we showed that that Delta and Omicron spikes were recognized by MBL to the same extent as the ancestral spike, and that binding to spike resulted in lectin pathway activation. At the same time, binding of serum MBL to spike was severely compromised after vaccination, likely because high-affinity antibodies, in concentrations several orders of magnitude higher than MBL, are displacing it from the virus antigen. This antibody displacement may also explain the disparity in the literature regarding MBL levels and COVID-19 severity. MBL recognized full-length spike ectodomain and NTD, but not the RBD, in agreement with the limited N-glycosylation of the latter. Removal of N-glycans sites at positions 61 in the NTD, as well as 603, 709, and 1074 in the S1/S2 domain by site-directed mutagenesis resulted in minor, non-statistically significant reductions in MBL binding in our experimental setup. Overall, none of the 12 examined glycan positions were critical for MBL recognition. We propose this is due to the flexibility of the MBL molecule that allows it to reach and bind any glycosylated position with hexoses with equatorial hydroxyl groups in the end position. Of interest, we observed a marked decrease in protein yield when expressing recombinant spike protein containing the N717Q and N801Q mutations, as well as altered denaturation profiles, and extra bands by SDS-PAGE suggestive of protein processing or degradation. N-linked glycans are known regulators of protein folding and quality control in the endoplasmic reticulum (90). Glycans in the 717 and 801 position, in close proximity in the S2 domain, appear to be important for protein folding and as such, we do not expect any future SARS-CoV-2 variants to fix these mutations.

We also monitored the evolution of IgG, IgA, and nAb titers in a cohort of vaccinees who were either infection-naïve, or had experienced a SARS-CoV-2 wt, Delta, or Omicron infection. In agreement with others, we showed that while antibody titers increase after vaccination or after breakthrough infections, titers plateau by three antigen exposures (i.e. three vaccine doses or two doses and infection) (54, 91). We also observed that the decline in titers was more pronounced for IgA than IgG. Moreover, we demonstrated that antibody avidity also plateaued by three antigen exposures. While many studies have looked at the waning of antibody levels over time, literature about the affinity maturation of SARS-CoV-2 antibodies after infection and vaccination remain sparse (92). Here we showed that hybrid immunity enhanced not only antibody titers, but also affinity maturation, specially of the IgA isotype. While most hybrid immune individuals developed measurable IgA responses, only 30–40% of the infection-naïve did so. It is now well established that most COVID-19-recovered individuals develop IgM, IgG, and IgA antibodies within the first two weeks after symptom onset (93–102), and that SARS-CoV-2 vaccination induces IgM and IgA responses, albeit not in all individuals and of a more transient nature than IgG responses (103–112). Nevertheless, the contribution of systemic serum IgA in the protection against SARS-CoV-2 infection remains unclear. IgA dominates the neutralizing response in the first two weeks after symptom onset (113, 114), apparently thanks to the enhanced flexibility of its hinge (114, 115). However, it should be kept in mind that IgG antibodies have not undergone sufficient affinity maturation in such early period after infection, as demonstrated in our longitudinal affinity measurements. Furthermore, depletion of IgA or IgG in hybrid immune individuals indicated that most of the neutralizing potency of sera comes from IgG. Our results are in line with a recent publication showing that IgA responses have modest neutralizing activities (116), and as IgA levels wane more rapidly than IgG levels, the protective role of serum IgA against SARS-CoV-2 infection would likely be short-lived (116, 117).

In a recent letter describing the results of two open-label, nonrandomized clinical studies, the authors reported that a fourth monovalent booster dose has a minimal protective effect against infection with Omicron, ranging from 30% gains for the Pfizer-BioNTech vaccine to 11% for Moderna (118). Thus, one may think that booster usefulness is, at least in their current form, limited to transiently recover waning antibody levels, and not significantly enhance their neutralization potency or recognition breadth (119). This emphasizes the need of novel vaccines, capable of preventing infection against novel variants. Moreover, concerns have been raised regarding frequent boosters and their role in the generation of novel SARS-CoV-2 variants via breakthrough from vaccine-elicited immunity (120). We and others have proposed that exposure of the airways to the pathogen, whether through natural infection or nasal vaccination, may be required to generate mucosal immunity capable of preventing infection (i.e. sterilizing immunity) (105, 121–125). However, the disappointing results of two of the first Phase I clinical trials of intranasal vaccination—which failed to elicit robust mucosal or systemic immune responses— serve as a reminder of the challenges ahead towards making reliable nasal vaccines (126, 127).

Next, we evaluated the evasion of nAbs by the Delta and Omicron variants after one, two, and three vaccine doses in individuals with different infection histories. Overall, our findings are in good agreement with the current literature, where the Delta variant is anywhere from 2.5- to 9-fold less sensitive to vaccine-elicited nAbs (3, 8, 128, 129). It has been proposed that Delta mutations in the NTD greatly contribute to the variant’s resistance to immune sera (27). Similarly, it has now been extensively shown that BA.1 exhibits a potent immune evasion capacity when challenged with mAbs and sera from convalescent and vaccinated individuals (62, 66, 73, 129–137). This could possibly be the result of nAb reactivities clustering in the RBD, which is highly mutated in the BA.1 variant (7). Many others have also looked at the neutralization of the BA.2 sublineage, which for the most part appears to be neutralized more efficiently than BA.1 (130, 138–140), or at least to a comparable extent (141–143). Direct comparisons of the neutralization potency of sera, after correcting for the number of exposures to the antigen (either as vaccine or infection), demonstrated that those with hybrid immunity mount stronger antibody responses than infection-naïve individuals. Thus, our data underscores the concept that hybrid immunity confers a more robust protection against infection, proposed to be the result of a broader and more sustained immune response due to the recognition of antigens not included in the spike-based vaccines, as well as the induction of mucosal immunity (144, 145). However, infection does come with risks (several orders of magnitude higher than vaccine-related risks) and we would like to discourage anyone from seeking this enhanced protection willingly.

Finally, we analyzed the impact of Delta and Omicron BA.1 on cellular immunity by measuring released cytokines after whole blood stimulation with pools of peptides spanning the entire length of the spike protein. IFN-γ is one of the main cytokines released during infection by cytotoxic (CD8) and Th1 helper (CD4) T cells, and induces an antiviral state by promoting differentiation and proliferation of T and B cells, and activation of phagocytes (146). By using peptides covering the spike or the remainder of the proteome excluding the spike, we could differentiate between vaccinated individuals with a previous infection and those reportedly never infected (specificity 100%, sensitivity 84.21%/92.31% for wt infection or Omicron infection, respectively). Unfortunately, we could not include non-vaccinees due to the high vaccination rates in Denmark. For such comparisons, we refer to the original publication describing these reagents (42). We also show a statistically significant reduction in released IFN-γ after stimulation with peptide pools from the spike Delta and Omicron, suggesting that some of the T cell epitopes are affected by the mutations in the new strains. However, and in contrast with the antibody responses, T cell responses remain largely unaffected. Our findings are in line with recent reports highlighting the relatively preserved T cell responses against the Omicron variant (51, 52, 64, 147, 148). The broad CD4+ and CD8+ T cell reactivity across the whole length of the spike protein, as well as the tolerance for substitutions in peptides presented by MHC molecules–due to the intrinsic flexibility of the latter (149)—may explain why T cell responses are not severely impaired by the heavily mutated Delta and Omicron spikes. Yet, as novel Omicron variants are identified across the globe, T cell responses should continue to be monitored for any signs of immune escape.

These conclusions must be considered in light of the limitations of the current study. The MS analyses of the glycan shields of the spike proteins are limited when it comes to the depth of the analyses and sample processing. Trypsin digestion, instead of using multiple distinct proteases, left ~30% of the sites unmapped, and automated glycan occupancy would benefit from a time-intensive manual curation. Still, it is reassuring to confirm that our findings are in agreement with other reports (25, 150). MS and biochemical data were derived from soluble recombinantly produced spike and RBD proteins, and not spikes presented on viral particles. Similarly, measurements of nAbs were done using antibody-mediated ACE-2/RBD inhibition ELISAs instead of using life viruses in plaque reduction neutralization tests. However, we have shown in the past that results from these tests are highly correlated (*ρ* = 0.9231, *p* < 0.0001) (41). Longitudinal blood samples were taken, on average, three weeks after the first dose, one month after the second dose, and two months after the third dose, and thus, the antibodies after the third dose may have waned slightly over that extra month from vaccine to sampling. Females are overrepresented in our cohort (88.5%), which reflects the sex imbalance of healthcare personnel in Denmark (our donor group), but we observed no statistically significant difference between vaccination/infection groups. Moreover, comparisons on the neutralizing potency of sera were only possible among those with a wt infection, because samples from Delta and Omicron convalescent individuals were taken after three vaccine doses plus a recent infection, and thus the contribution from each cannot be untangled. We focused on IgA titers in serum and their contribution to neutralization. However, IgA is mainly found in the mucosa and the circulating IgA levels might not reflect the actual levels in the respiratory tract. Finally, immune imprinting of cellular responses was evaluated using peptide mixes, where the contribution from *de novo* responses may be masked by the loss reactivity towards epitopes that are lost with the variant mutations. Finer analyses at the epitope level would be necessary to disentangle these counteracting responses.

In conclusion, here we demonstrated that the SARS-CoV-2 Delta and Omicron VOCs present marked gain in ACE-2 affinity and immune evasion, in particular the Omicron BA.1 sub-lineage. However, these immune evasive gains are predominantly limited to antibody responses, as recognition by the innate pattern recognition molecule MBL and T cell responses remain largely unaffected. MBL recognition appeared to be outcompeted by antibodies from vaccinee sera, in line of the conventional view of innate immunity as first line responders vying for time until the body can mount an adaptive immune response. Moreover, we provide insight into the impact of infection in antibody maturation, and how immune imprinting from vaccines formulated with the ancestral strain may limit the breath and compromise the efficacy of antibody and cellular responses against SARS-CoV-2 variants.

## Materials and methods

### Recombinant proteins

The nucleotide sequence of the spike ectodomain (amino acid [aa] 1–1208) of the SARS-CoV-2 Delta (Pango lineage B.1.617.2) and BA.1 Omicron strains (B.1.1.529), containing two proline substitutions at residues 986–987 and a GSAS substitution at residues 682–685 (numbering according to the wt spike), were synthesized by GeneArt (Thermo Fisher Scientific, Waltham, MA, USA) in the pcDNA3.4 expression vector. The Omicron spike sequence was followed by an 8xHis tag and the Delta spike by a T4 fibritin oligomerization domain and an 8xHis tag. Similarly, the sequences for the Delta, Omicron BA.1, and Omicron BA.2 RBDs (aa 319–591, numbering according to the wt) were synthesized by GeneArt with an N-terminal human serum albumin signal peptide and a C-terminal tandem 8xHis-Avi tag. The SARS-CoV-2 N-terminal region (aa 27–305) was synthesized with a CD33 secretion signal peptide (MPLLLLLPLLWAGALA) followed by an N-terminal 6xHis tag. These proteins, as well as the SARS-CoV-2 RBD and spike wt, protein N; and human ACE-2, ACE-2-Fc, and MBL were produced and purified as described previously (38, 41, 102, 151). The spike glycan variants were generated by site-directed mutagenesis by GeneArt (Thermo Fisher Scientific). Selected glycan variants were batch-purified by immobilized metal affinity chromatography (IMAC). Briefly, ExpiCHO supernatants (50 ml) were centrifuged at 1000 x *g* for 5 min at 4°C and filtered through 0.45 µm PVDF syringe filters (SLHV033RS Millipore/Merck, Rahway, NJ, USA). Clarified supernatants were diluted 1:2 with equilibration buffer (20 mM sodium phosphate, 150 mM sodium chloride), and incubated with 0.6 ml of HisPur Ni-NTA agarose beads (Thermo Fisher Scientific) for 2 h with end-over-end rotation at room temperature (RT). Bound proteins were eluted using equilibration buffer + 250 mM imidazole, buffer exchanged in PBS, and concentrated using Amicon filters with a 50 kDa cut-off (Merck). The following recombinant proteins were purchased from ACROBiosystems (Newark, NJ, USA): SARS-CoV-2 RBD BA.1 (SPD-C522e), RBD BA.2 (SPD-C522g), spike wt (SPN-C52H9), spike Delta (SPN-C52He), spike BA.1 (SPN-C52Hz), spike BA.2 (SPN-C5223), biotinylated RBD BA.1 (SPD-C82E4), and biotinylated RBD BA.2 (SPD-C82Eq). Additionally, a control SARS-CoV-2 trimeric Spike (101007) was provided by the NIBSC Repository, UK, with thanks to Dr Barney Graham, NIAID. This protein is identical to our spike wt with the addition of a C-terminal HRV 3C cleavage site (LEVLFQGPG) and two copies of the Strep-tag-II separated by a Gly-Ser linker (SAWSHPQFEKGGGSGGGGSGGSAWSHPQFEK).

### ACE-2 binding kinetics determination by biolayer interferometry

Kinetic measurements of the RBD wt, Delta, BA.1, and BA.2 interaction with ACE-2 were performed by BLI in an Octect RED383 system (ForteBio, Fremont, CA, USA) as described previously (20), only updating the BLI running buffer (1xPBS [AM9624 Invitrogen, Thermo Fisher Scientific], 0.1% bovine serum albumin (BSA) IgG free [A0336 Sigma-Aldrich, St. Louis, MO, USA], 0.03% Tween 20, pH 7.4) and the RBD variants being evaluated. Briefly, an ACE-2-Fc fusion protein (38) (13 µg/ml) was immobilized onto anti-human Fc capture sensors (Pall Life Sciences, California, USA) (500 s), followed by a baseline step (60 s), an association step (500 s) by dipping the sensors in 12-point, 1.5-fold serial dilutions of RBD wt, Delta, BA.1, and BA.2 (starting concentration 150 nM), and a final dissociation step (500 s). Sensorgrams were reference subtracted (ACE-2-Fc sensors in buffer only during the association and dissociation phases) and globally fitted to a 1:1 binding model.

### Thermal stability

Nano differential scanning fluorimetry (NanoDSF) was used to determine the impact of RBD-mapping mutations from the Delta, BA.1, and BA.2 strains, as well as the glycan mutations, on the stability of the RBD and the spike protein, respectively. Samples diluted in PBS (200 µg/ml) were analyzed in triplicates on a Tycho NT.6 (NanoTemper Technologies GmbH, Munich, Germany) on a predefined 30°C/min thermal ramp. The maxima in the first derivative of the ratio of the intrinsic fluorescence at 350 and 330 nm averaged from three replicates were used to calculate the inflection temperature (Ti), i.e. the temperature at which a discrete unfolding event takes place.

### Glycan analyses by mass spectrometry

Glycan profiles were assessed by fluorescence labelling of released N-glycans. A total of 50 µg of protein were treated with rapid PNGaseF (New England Biolabs, Ipswich, MA, USA) in order to release N-glycans. LC-MS analysis was performed on a Synapt G2Si Q-tof instrument (Waters Corp., Milford, MA, USA) using standard intact protein analysis settings and it was verified that all N-glycans had been removed. Subsequently fluorescence labelling of the released glycans was performed using GlycoWorks rapiFlour reagents and workflow (Waters Corp.). The labelled glycans were analysed by LC-MS and fluorescence detection using a Synapt G2Si Q-tof instrument with Acquity Premier UPLC inlet system and a Acquity UPLC Glycan BEH Amide, 100Å, 1.7 µm, 2.1 x 50 mm column for separation of labelled glycans (all from Waters Corp.). Ammonium Formate pH 4 and 100% MeCN were used as mobile phases A and B, respectively. A gradient of 25–46% buffer A over 35 min and a flow of 0.4 ml/min was employed.

Direct MS analysis of intact proteins was performed using a Ultraflex MALDI-TOF instrument (Bruker, Billerica, MA, USA). Samples were mixed 1:2 with sinapinic acid matrix and 1 µl applied to the target surface and allowed to dry.

### MBL spike interaction

#### Binding of native MBL from sera

MaxiSorp™ 96-well microtiter plates (439454 Thermo Fisher Scientific) were coated with 2 µg/ml of the spike variants, mannan (M7504 Sigma-Aldrich) as a positive control, and BSA (107350860001 Roche Diagnostics) as negative control, overnight (ON) at 4°C in PBS. MBL-containing serum samples diluted 1:3 in Barbital-T (4 mM sodium barbital, 145 mM NaCl, 2.6 mM CaCl_2_, 2.1 mM MgCl_2_, 0.05% Tween-20, pH 7.4) were added to the plates for 2 h. Bound MBL was detected with 2 µg/ml of biotinylated Hyb-131-1 (BioPorto Diagnostics, Hellerup, Denmark) for 1.5 h, followed by a 1:2000 dilution of streptavidin-HRP conjugate (RPN1231V Cytiva, Marlborough, MA, USA) for 1 h. Homozygous MBL-defect serum was used as negative control (152). Plates were developed with TMB ONE (KemEnTec Diagnostics, Taastrup, Denmark). The reaction was stopped with 0.3 M H_2_SO_4_, and the optical density (OD) was recorded at 450 – 630 nm. Plates were washed thrice between steps with Barbital-T. Incubations took place at RT on an orbital shaker.

#### Binding of recombinant MBL

Microtiter plates were coated with 2-fold dilutions, starting at 2.5 µg/ml of the spike variants, mannan as positive control, and BSA as negative control, ON at 4°C in PBS. A total of 0.5 µg/ml of rMBL in Barbital-T was added to the plates and allowed to bind for 2 h. Calcium dependency was evaluated on coated spike, mannan, and BSA (2 µg/ml), with rMBL diluted to 0.5 µg/ml in Barbital-T ± 10 mM EDTA and allowed to bind for 2 h. Detection and development were performed as described above. Incubations took place at RT on an orbital shaker.

#### MBL-mediated complement deposition

Microtiter plates were coated with 2 µg/ml of spike wt, mannan, and BSA ON at 4°C in PBS. A 3-fold dilution, starting at 5 µg/ml of rMBL in Barbital-T was added to the plates and incubated for 2 h. Next, MBL-defect serum (2% in Barbital-T) was incubated for 30 min (for C4 detection), 45 min (C3), and 60 min (TCC) at 37°C. Anti-C4 Hyb-162-02 (Bioporto), biotinylated anti-C3 BH6 (153), and anti-TCC aE11 (154) (all 2 µg/ml) were used as detection antibodies for 1.5 h, followed by polyclonal rabbit anti-mouse-HRP conjugate (P0260 Dako, Agilent, Santa Clara, CA, US) or streptavidin-HRP conjugate (both 1:2000 dilution) for 1 h. Plates were developed as described above. Plates were washed thrice between steps with Barbital-T. Unless otherwise stated, incubations took place at RT on an orbital shaker.

#### Binding of rMBL to spike glycan mutants

The spike glycan variants were produced in ExpiCHO cells (Thermo Fisher Scientific) using 24 deep well blocks (AXYGP-DW10ML24CS Corning Life Science, Tewksbury, MA, USA), and quantified by S-ELISA using *in-house* anti-spike antibodies (clone 53 and biotinylated clone 53 (41), both at 2 µg/ml). To evaluate the binding of rMBL to spike, microtiter plates were coated with 2 µg/ml of anti-spike clone 53 ON at 4°C in PBS. Supernatants (0.5 µg/ml) were incubated in TBS-T (10 mM Tris, 150 mM NaCl, 0.05% Tween-20, pH 7.4) supplemented with either 2.5 mM CaCl_2_ (calcium sufficient buffer) or 5 mM EDTA (calcium deficient) for 1.5 h. Next, rMBL (0.5 µg/ml in either calcium-sufficient or calcium-deficient buffer) was added to the plates and allowed to bind for 2 h. Detection, development, and overall handling were performed as described above.

### Blood samples

Humoral and cellular responses were evaluated longitudinally in serum and full-blood samples from healthy donors with or without a reverse transcription-polymerase chain reaction (RT‐PCR)-confirmed SARS-CoV-2 infection that received the BNT162b2 (Pfizer-BioNTech) vaccine. The participant cohort has been described in detail elsewhere (105, 155, 156), and a subset of them were randomly selected for this study (described in **Table 1**). For antibody analyses, samples were collected before the first vaccination, three weeks after the first dose (median 22 days, range 18–32), one month after the second dose (median 31 days, range 10–53), and two months after the third dose (median 83 days, range 22–207) (i.e. baseline, 3 weeks, 2 months, and 12 months after the first dose) (*n* = 80 for each group). Infection history was determined by RT-PCR and by the presence of anti-protein N antibodies determined by using the Elecsys^®^ Anti-SARS-CoV-2 assay (Roche Diagnostics, Basel, Switzerland) as described in our previous work (155). Donors were grouped as naïve (no reported positive RT-PCR test, and negative for antibodies against protein N), wt infection (positive RT-PCR test and protein N positive before the first sample collection), Delta infection (positive RT-PCR test only in the period between the end of August 2021 until the beginning of December 2021, and protein N negative until the last sample collection), and Omicron infection (positive RT-PCR test only in the period between end December and last sample collection, and protein N negative until the last sample collection). Venous blood samples were obtained after written and oral consent. Collection fulfilled the principles described in the Declaration of Helsinki and was approved by the Regional Scientific Ethics Committee of the Capital Region of Denmark (H-20079890).

### Affinity determination of a panel of murine mAbs towards the RBD variants

The effect of RBD mutations in the binding affinity of a panel of murine mAbs (*in-house*) (41) was evaluated by ELISA. Briefly, microtiters plates were coated with 2-fold dilutions of RBD wt, Delta, BA.1, and BA.2 (starting concentration 2 µg/ml) in PBS ON at 4°C. Murine mAbs antibodies (2 µg/ml) were added to the coated plates for 1.5 h, followed by detection with rabbit anti-mouse-HRP conjugate (P0260 Agilent) in a 1:2000 dilution. Plates were developed as described above. Unless otherwise stated, all incubations and washing steps took place at RT with PBS-T (0.05% Tween-20).

### Avidity maturation analyses

The evolution of IgG and IgA avidity was evaluated after the first, second, and third BNT162b2 vaccine dose in serum samples from randomly selected infection-naïve individuals, individuals infected before the first vaccine dose, putatively infected with Delta, and putatively infected with Omicron (*n* = 5 for all groups). IgG and IgA avidity was determined using the direct ELISA setup described elsewhere with the following modifications (102). Serum samples diluted in sample buffer were incubated in parallel in two RBD-coated (1 µg/ml) microtiter 96-well plates for 1 h. Next, plates were washed and incubated with either PBS-T or PBS-T + 5.5 mM urea (Amresco/VWR, Radnor, PA, USA) for 20 min. Detection of bound IgG and IgA was performed as described in the original protocol. Avidity was calculated as:


$$\text{Avidity }\left(\%\right)=\frac{\text{OD urea treated sample}}{\text{OD untreated sample}}x 100$$

### Antibody-mediated ACE-2/RBD inhibition

The potency of sera to neutralize the virus was assessed using a previously reported ACE-2/RBD inhibition assay with the following changes (41). Biotinylated RBD or spike wt, Delta, BA.1, and BA.2 were incubated with High Sensitivity streptavidin-HRP (21130; Thermo Fisher Scientific) (1:16000 dilution) and 6-point, 3-fold serial dilutions of vaccinee sera (starting dilution 10%). Alternatively, a 6-point, 4-fold dilution of murine monoclonal antibodies (mAbs) starting at 20 µg/ml was used instead of sera. The mAbs were raised by immunization of outbred NMRI mice with the spike ectodomain or RBD of the ancestral SARS-CoV-2 strain, and have been described elsewhere (41).

The RBD and spike variants concentration was determined from ACE-2 binding curves, choosing the point at the upper end of the linear region (ensuring the widest dynamic range when evaluating inhibition). Neutralizing potency of sera, reported as international units (IU)/ml, was calculated using the Working Reagent for anti-SARS-CoV-2 immunoglobulin 21/234 (NIBSC, Hertfordshire, UK) as standard. The threshold of positivity was determined based on the receiver operating characteristic (ROC) curves of each RBD variant from non-infected, non-vaccinated individuals (*n* = 50) and non-infected individuals after three BNT162b2 vaccine doses (*n* = 50). Sensitivity and specificity were 100% and 100% for wt (threshold 2382 IU/ml); 100% and 100% for Delta (threshold 1253 IU/ml); 100% and 98% for BA.1 (threshold 325.7 IU/ml); and 100% and 98% for BA.2 (threshold 279.3 IU/ml).

### IgG and IgA depletions

To discriminate between the contribution of IgG and IgA to the viral neutralization capacity of immune sera/plasma, we collected heparin plasma from five adult blood donors with a recent Omicron infection (median time from infection = 8 days, range 7–33) and depleted them for total IgG and/or total IgA.

Before depletion, the plasma was filtered through a 0.22 μm PVDF syringe filter (SLGV033RS Millipore/Merck). For depletion of IgG, the plasma was passed through a HiTrap Protein G HP 5 ml column (Cytiva) using Protein G binding buffer as washing buffer (20 mM NaH_2_PO_4_, pH 7). IgA was depleted with Peptide M agarose (InvivoGen, San Diego, CA, USA) using a Na_3_PO_4_ washing buffer (10 mM Na_3_PO_4_/150 mM NaCl, pH 7.2). For double depletion of IgG and IgA, the IgG depleted plasma was passed through the Peptide M column as described above.

Plasma samples depleted for IgG, IgA, and IgG+IgA were evaluated for the presence of nAbs in our antibody-mediated ACE-2/RBD inhibition assay as described above. Non-depleted samples were used as control.

### T cell stimulation in whole blood and cytokine release measurements

Blood samples from infection-naïve (*n* = 11), individuals with a previous infection (*n* = 6), and with an Omicron infection (*n* = 14) were collected by venipuncture in heparin tubes. Full-blood samples (0.5 ml) were stimulated with four different peptide mega pools (MP) (1 µg/ml in DMSO), described elsewhere (42, 43), for 21 h at 37°C. We used a SARS-CoV-2 spike wt MP containing 253 15-mer peptides, with a 10 aa overlap, to ensure complete coverage of the spike protein, as well as the spike Delta and Omicron BA.1 MP counterparts. Moreover, to discriminate between infection-naïve and those with a previous SARS-CoV-2 infection, we used a pool of experimentally defined MP specific for CD4 T cell responses comprising 284 15–20-mer peptides mapping to areas of the proteome outside the spike (CD4RE) (44). An equal volume of sterile DMSO was used as negative control. CFEX Ultra SuperStim pool (PM-CEFX-1 JPT, Berlin, Germany) (1 µg/ml) was used as positive control. After stimulation, samples were centrifuged at 2000 x *g* for 15 min, and plasma was collected and stored at −80°C for further analyses. IFN-γ release was measured using the Quant-T-cell ELISA kit (EQ 6841-9601 Euroimmun, Lübeck, Germany) in a cross-sectional cohort (*n* = 31), and a smaller longitudinal cohort of vaccinees with a recent Omicron infection (*n* = 9) followed for eight months after infection. The threshold of positivity was determined based on the ROC curves from vaccinated, non-infected individuals (*n* = 10), and vaccinated with a previous infection (any infection, likely wt, Alpha, and Omicron) (*n* = 19), or vaccinated with a recent Omicron infection (*n* = 13). Note that the latter group is included in the “any infection” group. Additionally, a human cytokine 27-plex assay (<ns/>M500KCAF0Y, Biorad, Hercules, CA, USA) was used to study the profile of released cytokines in a randomly selected cohort of vaccinees infection-naïve (*n* = 8) and convalescent (*n* = 14, 4 of them before and after Omicron infection) after stimulation with peptides MP of spike wt, CD4RE, and DMSO. The cytokine panel was analyzed with the Luminex 200 platform (R&D Systems, Minneapolis, MN, USA). Values below the detection limit were normalized to the lower limit of quantification interpolated from the standard for each cytokine.

### Murine model of heterologous prime-boost vaccination

To study the effect of immune imprinting in antibody and T cell responses, we measured antibody titers, nAbs, and T cell responses in a murine model for heterologous prime-boost vaccination. Animals’ care was in accordance with institutional guidelines, and the experimental procedures have been approved by the Danish Animal Experiments Inspectorate. Outbred female NMRI mice were divided into three groups: Wt (homologous vaccination, three doses of spike wt), Delta (heterologous, two doses of spike wt followed by a boost with spike Delta, n = 4), and Omicron (heterologous, two doses of spike wt followed by a boost with spike Omicron) (*n* = 4 for all groups). Mice were immunized thrice subcutaneously with 15 µg of spike adsorbed to GERBU P adjuvant (Gerbu, Heilderberg, Germany) as per manufacturer’s recommendation, with each immunization spaced 15 days apart. Blood samples were collected from the tail vein 14 days after each immunization, centrifuged at 2000 x *g* for 10 min at RT, diluted 1:10 in PBS, and stored at –20°C for later analyses. Mice were euthanized 15 days after the third immunization and had their spleens harvested. Spleens were homogenized, and splenocyte solutions were washed twice with complete RPMI media (RPMI 1640 [21870076], 1 X Penicillin-Streptomycin [15140122], 2 mM L-glutamine [A2916801], 1 mM sodium piruvate [11360070], 10% fetal bovine serum [26140079], all from Gibco/Thermo Fisher Scientific) by centrifugation at 350 x *g* for 10 min at 4°C using the shortest acceleration time/braking time. The supernatants were discarded, and the cells were resuspended by gentle pipetting in ice-cold ACK and incubated for 5 min at RT with occasional shaking. Lysis reactions were stopped with of complete RPMI medium, and washed twice as described previously. Splenocytes were counted on a Celldrop BF automated cell counter (DeNovix Inc., Wilmington, DE, USA) using trypan blue stain. Cell suspensions were diluted to 8 x 10^6^ viable cells/ml in complete RPMI media, and transferred to 24-well plates (500 µl/well, 4 x 10^6^ cells/well).

Splenocytes were stimulated with 1 μg/ml of peptide MP covering the spike wt, Delta, and Omicron for 72 h at 37°C in a humidified atmosphere with 5% CO_2_. An equal volume of DMSO was used as negative control. Supernatants were collected after centrifugation at 2000 x *g* for 10 min at 4°C and stored at –80°C for later analyses. IFN-γ release was measured in supernatants (1:25 dilution) using the IFN-γ Mouse Uncoated ELISA Kit (88-7314-22 Invitrogen).

### Statistics

Statistical analyses were performed using GraphPad Prism version 9.5 (GraphPad Software Inc, San Diego, California, USA). Comparisons of the binding of MBL to spike variants were done by Friedman tests with Dunn’s multiple comparisons. Statistical differences between antibody avidity of infection-naïve and hybrid immune individuals were evaluated by multiple Mann-Whitney tests with the two-stage linear step-up procedure Benjamini, Krieger, and Yekutieli. Differences in the neutralizing potency between RBD variants and depleted antibody isotypes, as well as between groups (infection) and vaccine doses were evaluated with two-way ANOVA with the Geisser-Greenhouse correction. The effect of prior infection on nAbs was analyzed by multiple Mann-Whitney tests with a two-stage linear step-up procedure of Benjamini, Krieger, and Yekutieli. Differences in the fold-change in neutralization of the RBD variants were evaluated using Kruskal-Wallis with Dunn’s multiple comparisons test. Outliers were identified using ROUT with Q = 1%. The affinity of murine mAbs (expressed as KD) towards the RBD variants was determined using the equation specific binding with Hill slope, while their neutralization potency (expressed as IC_50_) was calculated using the equation inhibitor concentration vs normalized response with variable response. Differences in affinity and neutralization potency were compared with the RBD wt by multiple Friedman tests with Dunn’s multiple comparisons correction. T cell responses were evaluated by multiple Kruskal-Wallis, Friedman tests with Dunn’s multiple comparisons corrections, and two-way ANOVA with the Geisser-Greenhouse correction. Half-life of T cell responses after spike and CD4RE stimulation were calculated using the one phase decay equation with least squares fit and constraining the plateau to 0. Differences in half-life were evaluated by Wilcoxon matched-pairs signed rank test. Correlation between antibodies or secreted cytokines were assessed by two-tailed Spearman rank’s correlation coefficient, with *p* values below 0.05 considered significant.

## Data Availability

The original contributions presented in the study are included in the article/**Supplementary Material**. Further inquiries can be directed to the corresponding author.

## References

[1] Jackson CB Farzan M Chen B Choe H. Mechanisms of SARS-CoV-2 entry into cells. Nat Rev Mol Cell Biol. (2022) 23:3–20. doi: 10.1038/s41580-021-00418-x 34611326 PMC8491763

[2] Harvey WT Carabelli AM Jackson B Gupta RK Thomson EC Harrison EM. SARS-CoV-2 variants, spike mutations and immune escape. Nat Rev Microbiol. (2021) 19:409–24. doi: 10.1038/s41579-021-00573-0 PMC8167834 34075212

[3] Mlcochova P Kemp SA Dhar MS Papa G Meng B Ferreira IATM. SARS-CoV-2 B.1.617.2 Delta variant replication and immune evasion. Nature. (2021) 599:114–9. doi: 10.1038/s41586-021-03944-y PMC8566220 34488225

[4] Cherian S Potdar V Jadhav S Yadav P Gupta N Das M. SARS-coV-2 spike mutations, L452R, T478K, E484Q and P681R, in the second wave of COVID-19 in maharashtra, India. Microorganisms. (2021) 9:1542. doi: 10.3390/microorganisms9071542 34361977 PMC8307577

[5] WHO. Tracking SARS-CoV-2 variants (2023). Available online at: https://www.who.int/en/activities/tracking-SARS-CoV-2-variants/ (Accessed 10 August 2022).

[6] Starr TN Greaney AJ Dingens AS Bloom JD. Complete map of SARS-CoV-2 RBD mutations that escape the monoclonal antibody LY-CoV555 and its cocktail with LY-CoV016. Cell Rep Med. (2021) 2:100255. doi: 10.1016/j.xcrm.2021.100255 33842902 PMC8020059

[7] Barnes CO Jette CA Abernathy ME Dam K-MA Esswein SR Gristick HB. SARS-CoV-2 neutralizing antibody structures inform therapeutic strategies. Nature. (2020) 588:682–7. doi: 10.1038/s41586-020-2852-1 PMC8092461 33045718

[8] Planas D Veyer D Baidaliuk A Staropoli I Guivel-Benhassine F Rajah MM. Reduced sensitivity of SARS-CoV-2 variant Delta to antibody neutralization. Nature. (2021) 596:276–80. doi: 10.1038/s41586-021-03777-9 34237773

[9] WHO. Classification of omicron (B.1.1.529): SARS-coV-2 variant of concern (2021). Available online at: https://www.who.int/news/item/26-11-2021-classification-of-omicron-(b.1.1.529)-sars-cov-2-variant-of-concern (Accessed August 10, 2022).

[10] Espenhain L Funk T Overvad M Edslev SM Fonager J Ingham AC. Epidemiological characterisation of the first 785 SARS-CoV-2 Omicron variant cases in Denmark, December 2021. Eurosurveillance. (2021) 26. doi: 10.2807/1560-7917.ES.2021.26.50.2101146 PMC8728489 34915977

[11] Brandal LT MacDonald E Veneti L Ravlo T Lange H Naseer U. Outbreak caused by the SARS-CoV-2 Omicron variant in Norway, November to December 2021. Euro Surveill. Bull Eur sur les Mal. Transm = Eur Commun Dis Bull. (2021) 26. doi: 10.2807/1560-7917.ES.2021.26.50.2101147 PMC8728491 34915975

[12] Maslo C Friedland R Toubkin M Laubscher A Akaloo T Kama B. Characteristics and outcomes of hospitalized patients in South Africa during the COVID-19 omicron wave compared with previous waves. JAMA. (2022) 327:583–4. doi: 10.1001/jama.2021.24868 PMC8719272 34967859

[13] Shuai H Chan JF-W Hu B Chai Y Yuen TT-T Yin F. Attenuated replication and pathogenicity of SARS-CoV-2 B.1.1.529 Omicron. Nature. (2022) 603:693–9. doi: 10.1038/s41586-022-04442-5 35062016

[14] Suzuki R Yamasoba D Kimura I Wang L Kishimoto M Ito J. Attenuated fusogenicity and pathogenicity of SARS-CoV-2 Omicron variant. Nature. (2022) 603:700–5. doi: 10.1038/s41586-022-04462-1 PMC8942852 35104835

[15] Lyngse FP Kirkeby CT Denwood M Christiansen LE Mølbak K Møller CH. Transmission of SARS-coV-2 omicron VOC subvariants BA.1 and BA.2: evidence from danish households. medRxiv. (2022) 2022:1.28.22270044. doi: 10.1101/2022.01.28.22270044

[16] Yamasoba D Kimura I Nasser H Morioka Y Nao N Ito J. Virological characteristics of the SARS-CoV-2 Omicron BA.2 spike. Cell. (2022) 185:2103–2115.e19. doi: 10.1016/j.cell.2022.04.035 35568035 PMC9057982

[17] Stegger M Edslev SM Sieber RN Cäcilia Ingham A Ng KL Tang M-HE. Occurrence and significance of Omicron BA.1 infection followed by BA.2 reinfection. medRxiv. (2022) 2022:2.19.22271112. doi: 10.1101/2022.02.19.22271112

[18] Hoffmann M Kleine-Weber H Schroeder S Krüger N Herrler T Erichsen S. SARS-coV-2 cell entry depends on ACE2 and TMPRSS2 and is blocked by a clinically proven protease inhibitor. Cell. (2020) 181:271–280.e8. doi: 10.1016/j.cell.2020.02.052 32142651 PMC7102627

[19] Piccoli L Park Y-J Tortorici MA Czudnochowski N Walls AC Beltramello M. Mapping neutralizing and immunodominant sites on the SARS-coV-2 spike receptor-binding domain by structure-guided high-resolution serology. Cell. (2020) 183:1024–1042.e21. doi: 10.1016/j.cell.2020.09.037 32991844 PMC7494283

[20] Bayarri-Olmos R Johnsen LB Idorn M Reinert LS Rosbjerg A Vang S. The alpha/B.1.1.7 SARS-CoV-2 variant exhibits significantly higher affinity for ACE-2 and requires lower inoculation doses to cause disease in K18-hACE2 mice. Elife. (2021) 10:e70002. doi: 10.7554/eLife.70002 34821555 PMC8635972

[21] Bayarri-Olmos R Jarlhelt I Johnsen LB Hansen CB Helgstrand C Rose Bjelke J. Functional effects of receptor-binding domain mutations of SARS-coV-2 B.1.351 and P.1 variants. Front Immunol. (2021) 12:4145. doi: 10.3389/fimmu.2021.757197 PMC8529273 34691078

[22] Health Security Agency UK. SARS-CoV-2 variants of concern and variants under investigation in England. Tech briefing 39. (2022), 41 p. Available online at: https://assets.publishing.service.gov.uk/government/uploads/system/uploads/attachment_data/file/1063424/Tech-Briefing-39-25March2022_FINAL.pdf.

[23] Garred P Genster N Pilely K Bayarri‐Olmos R Rosbjerg A Ma YJ. A journey through the lectin pathway of complement— MBL and beyond. Immunol Rev. (2016) 274:74–97. doi: 10.1111/imr.2016.274.issue-1 27782323

[24] Stravalaci M Pagani I Paraboschi EM Pedotti M Doni A Scavello F. Recognition and inhibition of SARS-CoV-2 by humoral innate immunity pattern recognition molecules. Nat Immunol. (2022) 23:275–86. doi: 10.1038/s41590-021-01114-w 35102342

[25] Newby ML Fogarty CA Allen JD Butler J Fadda E Crispin M. Variations within the glycan shield of SARS-coV-2 impact viral spike dynamics. J Mol Biol. (2023) 435:167928. doi: 10.1016/j.jmb.2022.167928 36565991 PMC9769069

[26] McCallum M De Marco A Lempp FA Tortorici MA Pinto D Walls AC. N-terminal domain antigenic mapping reveals a site of vulnerability for SARS-CoV-2. Cell. (2021) 184:2332–2347.e16. doi: 10.1016/j.cell.2021.03.028 33761326 PMC7962585

[27] Liu Y Arase N Kishikawa J Hirose M Li S Tada A. The SARS-CoV-2 Delta variant is poised to acquire complete resistance to wild-type spike vaccines. bioRxiv. (2021) 2021:8.22.457114. doi: 10.1101/2021.08.22.457114

[28] Plante JA Mitchell BM Plante KS Debbink K Weaver SC Menachery VD. The variant gambit: COVID-19’s next move. Cell Host Microbe. (2021) 29:508–15. doi: 10.1016/j.chom.2021.02.020 PMC7919536 33789086

[29] Plante JA Liu Y Liu J Xia H Johnson BA Lokugamage KG. Author Correction: Spike mutation D614G alters SARS-CoV-2 fitness. Nature. (2021) 595:E1–1. doi: 10.1038/s41586-021-03657-2 PMC8205212 34131306

[30] Hou YJ Chiba S Halfmann P Ehre C Kuroda M Dinnon KH. SARS-CoV-2 D614G variant exhibits efficient replication ex vivo and transmission. vivo. Sci (80-. ). (2020) 370:1464–8. doi: 10.1126/science.abe8499 PMC7775736 33184236

[31] McMillen T Jani K Robilotti EV Kamboj M Babady NE. The spike gene target failure (SGTF) genomic signature is highly accurate for the identification of Alpha and Omicron SARS-CoV-2 variants. Sci Rep. (2022) 12:18968. doi: 10.1038/s41598-022-21564-y 36347878 PMC9641688

[32] Gerdol M Dishnica K Giorgetti A. Emergence of a recurrent insertion in the N-terminal domain of the SARS-CoV-2 spike glycoprotein. Virus Res. (2022) 310:198674. doi: 10.1016/j.virusres.2022.198674 35021068 PMC8743576

[33] Lan J Ge J Yu J Shan S Zhou H Fan S. Structure of the SARS-CoV-2 spike receptor-binding domain bound to the ACE2 receptor. Nature. (2020) 581:215–20. doi: 10.1038/s41586-020-2180-5 32225176

[34] Zhang J Cai Y Lavine CL Peng H Zhu H Anand K. Structural and functional impact by SARS-CoV-2 Omicron spike mutations. Cell Rep. (2022) 39:110729. doi: 10.1016/j.celrep.2022.110729 35452593 PMC8995406

[35] Ozono S Zhang Y Ode H Sano K Tan TS Imai K. SARS-CoV-2 D614G spike mutation increases entry efficiency with enhanced ACE2-binding affinity. Nat Commun. (2021) 12:848. doi: 10.1038/s41467-021-21118-2 33558493 PMC7870668

[36] Yurkovetskiy L Wang X Pascal KE Tomkins-Tinch C Nyalile TP Wang Y. Structural and functional analysis of the D614G SARS-coV-2 spike protein variant. Cell. (2020) 183:739–751.e8. doi: 10.1016/j.cell.2020.09.032 32991842 PMC7492024

[37] Motozono C Toyoda M Zahradnik J Saito A Nasser H Tan TS. SARS-CoV-2 spike L452R variant evades cellular immunity and increases infectivity. Cell Host Microbe. (2021) 29:1124–1136.e11. doi: 10.1016/j.chom.2021.06.006 34171266 PMC8205251

[38] Bayarri-Olmos R Rosbjerg A Johnsen LB Helgstrand C Bak-Thomsen T Garred P. The SARS-CoV-2 Y453F mink variant displays a pronounced increase in ACE-2 affinity but does not challenge antibody neutralization. J Biol Chem. (2021) 296:100536. doi: 10.1016/j.jbc.2021.100536 33716040 PMC7948531

[39] Berger I Schaffitzel C. The SARS-CoV-2 spike protein: balancing stability and infectivity. Cell Res. (2020) 30:1059–60. doi: 10.1038/s41422-020-00430-4 PMC7604330 33139926

[40] Watanabe Y Allen JD Wrapp D McLellan JS Crispin M. Site-specific glycan analysis of the SARS-CoV-2 spike. Sci (80-. ). (2020) 369:330–3. doi: 10.1126/science.abb9983 PMC7199903 32366695

[41] Bayarri-Olmos R Idorn M Rosbjerg A Pérez-Alós L Hansen CB Johnsen LB. SARS-coV-2 neutralizing antibody responses towards full-length spike protein and the receptor-binding domain. J Immunol. (2021) 207:878–87. doi: 10.4049/jimmunol.2100272 34301847

[42] Yu ED Wang E Garrigan E Goodwin B Sutherland A Tarke A. Development of a T cell-based immunodiagnostic system to effectively distinguish SARS-CoV-2 infection and COVID-19 vaccination status. Cell Host Microbe. (2022) 30:388–399.e3. doi: 10.1016/j.chom.2022.02.003 35172129 PMC8824221

[43] Grifoni A Weiskopf D Ramirez SI Mateus J Dan JM Moderbacher CR. Targets of T cell responses to SARS-coV-2 coronavirus in humans with COVID-19 disease and unexposed individuals. Cell. (2020) 181:1489–1501.e15. doi: 10.1016/j.cell.2020.05.015 32473127 PMC7237901

[44] Grifoni A Sidney J Vita R Peters B Crotty S Weiskopf D. SARS-CoV-2 human T cell epitopes: Adaptive immune response against COVID-19. Cell Host Microbe. (2021) 29:1076–92. doi: 10.1016/j.chom.2021.05.010 PMC8139264 34237248

[45] Dhanda SK Vita R Ha B Grifoni A Peters B Sette A. ImmunomeBrowser: a tool to aggregate and visualize complex and heterogeneous epitopes in reference proteins. Bioinformatics. (2018) 34:3931–3. doi: 10.1093/bioinformatics/bty463 PMC6223373 29878047

[46] Garcia-Beltran WF Lam EC St. Denis K Nitido AD Garcia ZH Hauser BM. Multiple SARS-CoV-2 variants escape neutralization by vaccine-induced humoral immunity. Cell. (2021) 184:2372–2383.e9. doi: 10.1016/j.cell.2021.03.013 33743213 PMC7953441

[47] Richardson SI Madzorera VS Spencer H Manamela NP van der Mescht MA Lambson BE. SARS-CoV-2 Omicron triggers cross-reactive neutralization and Fc effector functions in previously vaccinated, but not unvaccinated, individuals. Cell Host Microbe. (2022) 30:880–886.e4. doi: 10.1016/j.chom.2022.03.029 35436444 PMC8947963

[48] Offit PA. Bivalent covid-19 vaccines — A cautionary tale. N Engl J Med. (2023) 388:481–3. doi: 10.1056/NEJMp2215780 36630616

[49] Wang Q Bowen A Valdez R Gherasim C Gordon A Liu L. Antibody response to omicron BA.4–BA.5 bivalent booster. N Engl J Med. (2023) 388:567–9. doi: 10.1056/NEJMc2213907 PMC9847504 36630643

[50] Collier AY Miller J Hachmann NP McMahan K Liu J Bondzie EA. Immunogenicity of BA.5 bivalent mRNA vaccine boosters. N Engl J Med. (2023) 388:565–7. doi: 10.1056/NEJMc2213948 PMC9847505 36630611

[51] Keeton R Tincho MB Ngomti A Baguma R Benede N Suzuki A. T cell responses to SARS-CoV-2 spike cross-recognize Omicron. Nature. (2022) 603:488–92. doi: 10.1038/s41586-022-04460-3 PMC8930768 35102311

[52] Gao Y Cai C Grifoni A Müller TR Niessl J Olofsson A. Ancestral SARS-CoV-2-specific T cells cross-recognize the Omicron variant. Nat Med. (2022) 28:472–6. doi: 10.1038/s41591-022-01700-x PMC8938268 35042228

[53] Jung MK Jeong SD Noh JY Kim D-U Jung S Song JY. BNT162b2-induced memory T cells respond to the Omicron variant with preserved polyfunctionality. Nat Microbiol. (2022) 7:909–17. doi: 10.1038/s41564-022-01123-x 35577972

[54] Reynolds CJ Pade C Gibbons JM Otter AD Lin K-M Muñoz Sandoval D. Immune boosting by B.1.1.529 (Omicron) depends on previous SARS-CoV-2 exposure. Sci (80-. ). (2022) 377:eabq1841. doi: 10.1126/science.abq1841 PMC9210451 35699621

[55] Kaku CI Bergeron AJ Ahlm C Normark J Sakharkar M Forsell MNE. Recall of preexisting cross-reactive B cell memory after Omicron BA. 1 breakthrough infection. Sci Immunol. (2022) 7:eabq3511. doi: 10.1126/sciimmunol.abq3511 35549299 PMC9097882

[56] Quandt J Muik A Salisch N Lui BG Lutz S Krüger K. Omicron BA.1 breakthrough infection drives cross-variant neutralization and memory B cell formation against conserved epitopes. Sci Immunol. (2022) 7:eabq2427. doi: 10.1126/sciimmunol.abq2427 35653438 PMC9162083

[57] Cao Y Jian F Wang J Yu Y Song W Yisimayi A. Imprinted SARS-CoV-2 humoral immunity induces convergent Omicron RBD evolution. Nature. (2022) 2022:09.15.507787. doi: 10.1038/s41586-022-05644-7 PMC9931576 36535326

[58] Kaku CI Starr TN Zhou P Dugan HL Khalifé P Song G. Evolution of antibody immunity following Omicron BA.1 breakthrough infection. Nat Commun. (2023) 14:2751. doi: 10.1038/s41467-023-38345-4 37173311 PMC10180619

[59] Sokal A Barba-Spaeth G Hunault L Fernández I Broketa M Meola A. SARS-CoV-2 Omicron BA.1 breakthrough infection drives late remodeling of the memory B cell repertoire in vaccinated individuals. Immunity. (2023) 56:2137–2151.e7. doi: 10.1016/j.immuni.2023.07.007 37543032

[60] Willett BJ Grove J MacLean OA Wilkie C De Lorenzo G Furnon W. SARS-CoV-2 Omicron is an immune escape variant with an altered cell entry pathway. Nat Microbiol. (2022) 7:1161–79. doi: 10.1038/s41564-022-01143-7 PMC9352574 35798890

[61] Li L Liao H Meng Y Li W Han P Liu K. Structural basis of human ACE2 higher binding affinity to currently circulating Omicron SARS-CoV-2 sub-variants BA.2 and BA.1.1. Cell. (2022) 185:2952–2960.e10. doi: 10.1016/j.cell.2022.06.023 35809570 PMC9212699

[62] Cameroni E Bowen JE Rosen LE Saliba C Zepeda SK Culap K. Broadly neutralizing antibodies overcome SARS-CoV-2 Omicron antigenic shift. Nature. (2022) 602:664–70. doi: 10.1038/s41586-021-04386-2 PMC9531318 35016195

[63] Moulana A Dupic T Phillips AM Chang J Nieves S Roffler AA. Compensatory epistasis maintains ACE2 affinity in SARS-CoV-2 Omicron BA.1. Nat Commun. (2022) 13:7011. doi: 10.1038/s41467-022-34506-z 36384919 PMC9668218

[64] Tarke A Coelho CH Zhang Z Dan JM Yu ED Methot N. SARS-CoV-2 vaccination induces immunological T cell memory able to cross-recognize variants from Alpha to Omicron. Cell. (2022) 185:847–859.e11. doi: 10.1016/j.cell.2022.01.015 35139340 PMC8784649

[65] Han P Li L Liu S Wang Q Zhang D Xu Z. Receptor binding and complex structures of human ACE2 to spike RBD from omicron and delta SARS-CoV-2. Cell. (2022) 185:630–640.e10. doi: 10.1016/j.cell.2022.01.001 35093192 PMC8733278

[66] Zhang X Wu S Wu B Yang Q Chen A Li Y. SARS-CoV-2 Omicron strain exhibits potent capabilities for immune evasion and viral entrance. Signal Transduction Targeting Ther. (2021) 6:430. doi: 10.1038/s41392-021-00852-5 PMC8678971 34921135

[67] Starr TN Greaney AJ Hilton SK Ellis D Crawford KHD Dingens AS. Deep mutational scanning of SARS-coV-2 receptor binding domain reveals constraints on folding and ACE2 binding. Cell. (2020) 182:1295–1310.e20. doi: 10.1016/j.cell.2020.08.012 32841599 PMC7418704

[68] Sonnleitner ST Prelog M Sonnleitner S Hinterbichler E Halbfurter H Kopecky DBC. Cumulative SARS-CoV-2 mutations and corresponding changes in immunity in an immunocompromised patient indicate viral evolution within the host. Nat Commun. (2022) 13:2560. doi: 10.1038/s41467-022-30163-4 35538074 PMC9090742

[69] Corey L Beyrer C Cohen MS Michael NL Bedford T Rolland M. SARS-coV-2 variants in patients with immunosuppression. N Engl J Med. (2021) 385:562–6. doi: 10.1056/NEJMsb2104756 PMC8494465 34347959

[70] Choi B Choudhary MC Regan J Sparks JA Padera RF Qiu X. Persistence and evolution of SARS-coV-2 in an immunocompromised host. New Engl J Med. (2020) 383:2291–3. doi: 10.1056/NEJMc2031364 PMC7673303 33176080

[71] Kemp SA Collier DA Datir RP Ferreira IATM Gayed S Jahun A. SARS-CoV-2 evolution during treatment of chronic infection. Nature. (2021) 592:277–82. doi: 10.1038/s41586-021-03291-y PMC7610568 33545711

[72] Greaney AJ Loes AN Crawford KHD Starr TN Malone KD Chu HY. Comprehensive mapping of mutations in the SARS-CoV-2 receptor-binding domain that affect recognition by polyclonal human plasma antibodies. Cell Host Microbe. (2021) 29:463–476.e6. doi: 10.1016/j.chom.2021.02.003 33592168 PMC7869748

[73] Cao Y Wang J Jian F Xiao T Song W Yisimayi A. Omicron escapes the majority of existing SARS-CoV-2 neutralizing antibodies. Nature. (2022) 602:657–63. doi: 10.1038/s41586-021-04385-3 PMC8866119 35016194

[74] Lin S Chen Z Zhang X Wen A Yuan X Yu C. Characterization of SARS-CoV-2 Omicron spike RBD reveals significantly decreased stability, severe evasion of neutralizing-antibody recognition but unaffected engagement by decoy ACE2 modified for enhanced RBD binding. Signal Transduction Targeting Ther. (2022) 7:56. doi: 10.1038/s41392-022-00914-2 PMC8860268 35190526

[75] Plante JA Liu Y Liu J Xia H Johnson BA Lokugamage KG. Spike mutation D614G alters SARS-CoV-2 fitness. Nature. (2020) 592:116–21. doi: 10.1038/s41586-020-2895-3 PMC8158177 33106671

[76] Peacock TP Brown JC Zhou J Thakur N Sukhova K Newman J. The altered entry pathway and antigenic distance of the SARS-CoV-2 Omicron variant map to separate domains of spike protein. bioRxiv. (2022) 2021:12.31.474653. doi: 10.1101/2021.12.31.474653

[77] Hui KPY Ho JCW Cheung M Ng K Ching RHH Lai K. SARS-CoV-2 Omicron variant replication in human bronchus and lung ex vivo. Nature. (2022) 603:715–20. doi: 10.1038/s41586-022-04479-6 35104836

[78] Watanabe Y Bowden TA Wilson IA Crispin M. Exploitation of glycosylation in enveloped virus pathobiology. Biochim Biophys Acta Gen Subj. (2019) 1863:1480–97. doi: 10.1016/j.bbagen.2019.05.012 PMC6686077 31121217

[79] Altman MO Angel M Košík I Trovão NS Zost SJ Gibbs JS. Human influenza A virus hemagglutinin glycan evolution follows a temporal pattern to a glycan limit. MBio. (2019) 10:e00204–19. doi: 10.1128/mBio.00204-19 PMC6445938 30940704

[80] Coss KP Vasiljevic S Pritchard LK Krumm SA Glaze M Madzorera S. HIV-1 glycan density drives the persistence of the mannose patch within an infected individual. J Virol. (2016) 90:11132–44. doi: 10.1128/JVI.01542-16 PMC5126371 27707925

[81] Medetalibeyoglu A Bahat G Senkal N Kose M Avci K Sayin GY. Mannose binding lectin gene 2 (rs1800450) missense variant may contribute to development and severity of COVID-19 infection. Infect Genet Evol J Mol Epidemiol. Evol Genet Infect Dis. (2021) 89:104717. doi: 10.1016/j.meegid.2021.104717 PMC7838598 33515713

[82] Speletas M Dadouli K Syrakouli A Gatselis N Germanidis G Mouchtouri VA. MBL deficiency-causing B allele (rs1800450) as a risk factor for severe COVID-19. Immunobiology. (2021) 226:152136. doi: 10.1016/j.imbio.2021.152136 34628288 PMC8462051

[83] Queiroz MAF Santiago AM Brito WRdS Pereira KAS de Brito WB Torres MKdS. Polymorphisms in the MBL2 gene are associated with the plasma levels of MBL and the cytokines IL-6 and TNF-α in severe COVID-19. Front Immunol. (2023) 14. doi: 10.3389/fimmu.2023.1151058 PMC10149935 37138871

[84] Pairo-Castineira E Rawlik K Bretherick AD Qi T Wu Y Nassiri I. GWAS and meta-analysis identifies 49 genetic variants underlying critical COVID-19. Nature. (2023) 617:764–8. doi: 10.1038/s41586-023-06034-3 PMC10208981 37198478

[85] Charitos P Heijnen IAFM Egli A Bassetti S Trendelenburg M Osthoff M. Functional activity of the complement system in hospitalized COVID-19 patients: A prospective cohort study. Front Immunol. (2021) 12:765330. doi: 10.3389/fimmu.2021.765330 34777382 PMC8581394

[86] Kashiwagi Y Suzuki S Takahashi R Yamanaka G Hirai Y Kawashima H. Association of the mannose-binding lectin 2 BB genotype with COVID-19-related mortality. Life. (2023) 13. doi: 10.3390/life13020382 PMC9961194 36836739

[87] Hultström M Frithiof R Grip J Lindelöf L Rooijackers O Pigazzini S. Genetic determinants of mannose-binding lectin activity predispose to thromboembolic complications in critical COVID-19. Nat Immunol. (2022) 23:861–4. doi: 10.1038/s41590-022-01227-w 35624204

[88] Holter JC Pischke SE de Boer E Lind A Jenum S Holten AR. Systemic complement activation is associated with respiratory failure in COVID-19 hospitalized patients. Proc Natl Acad Sci U. S. A. (2020) 117:25018–25. doi: 10.1073/pnas.2010540117 PMC7547220 32943538

[89] Asselta R Paraboschi EM Stravalaci M Invernizzi P Bonfanti P Biondi A. Reply to: Hultström et al., Genetic determinants of mannose-binding lectin activity predispose to thromboembolic complications in critical COVID-19. Mannose-binding lectin genetics in COVID-19. Nat Immunol. (2022) 23:865–7. doi: 10.1038/s41590-022-01228-9 35624207

[90] Tannous A Pisoni GB Hebert DN Molinari M. N-linked sugar-regulated protein folding and quality control in the ER. Semin Cell Dev Biol. (2015) 41:79–89. doi: 10.1016/j.semcdb.2014.12.001 25534658 PMC4474783

[91] Reynolds CJ Gibbons JM Pade C Lin K-M Sandoval DM Pieper F. Heterologous infection and vaccination shapes immunity against SARS-CoV-2 variants. Sci (80-. ). (2022) 375:183–92. doi: 10.1126/science.abm0811 PMC10186585 34855510

[92] Bellusci L Grubbs G Zahra FT Forgacs D Golding H Ross TM. Antibody affinity and cross-variant neutralization of SARS-CoV-2 Omicron BA.1, BA.2 and BA.3 following third mRNA vaccination. Nat Commun. (2022) 13:4617. doi: 10.1038/s41467-022-32298-w 35941152 PMC9358642

[93] Wang Y Zhang L Sang L Ye F Ruan S Zhong B. Kinetics of viral load and antibody response in relation to COVID-19 severity. J Clin Invest. (2020) 130:5235–44. doi: 10.1172/JCI138759 PMC7524490 32634129

[94] Zhao J Yuan Q Wang H Liu W Liao X Su Y. Antibody responses to SARS-CoV-2 in patients of novel coronavirus disease 2019. Clin Infect Dis an Off Publ. Infect Dis Soc Am. (2020) 71:2027–34. doi: 10.1093/cid/ciaa344 PMC7184337 32221519

[95] Robbiani DF Gaebler C Muecksch F Lorenzi JCC Wang Z Cho A. Convergent antibody responses to SARS-CoV-2 in convalescent individuals. Nature. (2020) 584:437–42. doi: 10.1038/s41586-020-2456-9 PMC7442695 32555388

[96] Okba NMA Müller MA Li W Wang C GeurtsvanKessel CH Corman VM. Severe acute respiratory syndrome coronavirus 2-specific antibody responses in coronavirus disease patients. Emerg Infect Dis. (2020) 26:1478–88. doi: 10.3201/eid2607.200841 PMC7323511 32267220

[97] Ma H Zeng W He H Zhao D Jiang D Zhou P. Serum igA, igM, and igG responses in COVID-19. Cell Mol Immunol. (2020) 17:773–5. doi: 10.1038/s41423-020-0474-z PMC7331804 32467617

[98] Long Q-X Liu B-Z Deng H-J Wu G-C Deng K Chen Y-K. Antibody responses to SARS-CoV-2 in patients with COVID-19. Nat Med. (2020) 26:845–8. doi: 10.1038/s41591-020-0897-1 32350462

[99] Guo L Ren L Yang S Xiao M Chang D Yang F. Profiling early humoral response to diagnose novel coronavirus disease (COVID-19). Clin Infect Dis an Off Publ. Infect Dis Soc Am. (2020) 71:778–85. doi: 10.1093/cid/ciaa310 PMC7184472 32198501

[100] Padoan A Sciacovelli L Basso D Negrini D Zuin S Cosma C. IgA-Ab response to spike glycoprotein of SARS-CoV-2 in patients with COVID-19: A longitudinal study. Clin Chim Acta. (2020) 507:164–6. doi: 10.1016/j.cca.2020.04.026 PMC7194886 32343948

[101] Prévost J Gasser R Beaudoin-Bussières G Richard J Duerr R Laumaea A. Cross-sectional evaluation of humoral responses against SARS-coV-2 spike. Cell Rep Med. (2020) 1:100126. doi: 10.1016/j.xcrm.2020.100126 33015650 PMC7524645

[102] Hansen CB Jarlhelt I Pérez-Alós L Hummelshøj Landsy L Loftager M Rosbjerg A. SARS-coV-2 antibody responses are correlated to disease severity in COVID-19 convalescent individuals. J Immunol. (2021) 206:109–17. doi: 10.4049/jimmunol.2000898 33208457

[103] Wang Z Schmidt F Weisblum Y Muecksch F Barnes CO Finkin S. mRNA vaccine-elicited antibodies to SARS-CoV-2 and circulating variants. Nature. (2021) 592:616–22. doi: 10.1038/s41586-021-03324-6 PMC8503938 33567448

[104] Ruggiero A Piubelli C Calciano L Accordini S Valenti MT Carbonare LD. SARS-CoV-2 vaccination elicits unconventional IgM specific responses in naïve and previously COVID-19-infected individuals. eBioMedicine. (2022) 77:103888. doi: 10.1016/j.ebiom.2022.103888 35196644 PMC8858081

[105] Pérez-Alós L Armenteros JJA Madsen JR Hansen CB Jarlhelt I Hamm SR. Modeling of waning immunity after SARS-CoV-2 vaccination and influencing factors. Nat Commun. (2022) 13:1614. doi: 10.1038/s41467-022-29225-4 35347129 PMC8960902

[106] Gobbi F Buonfrate D Moro L Rodari P Piubelli C Caldrer S. Antibody response to the BNT162b2 mRNA COVID-19 vaccine in subjects with prior SARS-coV-2 infection. Viruses. (2021) 13:422. doi: 10.3390/v13030422 33807957 PMC8001674

[107] Naaber P Tserel L Kangro K Sepp E Jürjenson V Adamson A. Dynamics of antibody response to BNT162b2 vaccine after six months: a longitudinal prospective study. Lancet Reg. Heal - Eur. (2021) 10:100208. doi: 10.1016/j.lanepe.2021.100208 PMC8418937 34514454

[108] Chan RWY Liu S Cheung JY Tsun JGS Chan KC Chan KYY. The mucosal and serological immune responses to the novel coronavirus (SARS-coV-2) vaccines. Front Immunol. (2021) 12. doi: 10.3389/fimmu.2021.744887 PMC8547269 34712232

[109] Takamatsu Y Omata K Shimizu Y Kinoshita-Iwamoto N Terada M Suzuki T. SARS-CoV-2-neutralizing humoral IgA response occurs earlier but modest and diminishes faster compared to IgG response. bioRxiv : preprint server Biol. (2022). doi: 10.1101/2022.06.09.495422 PMC9769934 36219096

[110] Scrimin F Campisciano G Comar M Ragazzon C Davanzo R Quadrifoglio M. IgG and igA antibodies post SARS-coV-2 vaccine in the breast milk and sera of breastfeeding women. Vaccines. (2022) 10:125. doi: 10.3390/vaccines10010125 35062786 PMC8778843

[111] Wisnewski AV Campillo Luna J Redlich CA. Human IgG and IgA responses to COVID-19 mRNA vaccines. PloS One. (2021) 16:e0249499. doi: 10.1371/journal.pone.0249499 34133415 PMC8208542

[112] Melgoza-González EA Hinojosa-Trujillo D Reséndiz-Sandoval M Mata-Haro V Hernández-Valenzuela S García-Vega M. Analysis of IgG, IgA and IgM antibodies against SARS-CoV-2 spike protein S1 in convalescent and vaccinated patients with the Pfizer-BioNTech and CanSinoBio vaccines. Transbound Emerg Dis. (2022) 69:e734–45. doi: 10.1111/tbed.14344 PMC8662108 34655457

[113] Kurano M Morita Y Nakano Y Yokoyama R Shimura T Qian C. Response kinetics of different classes of antibodies to SARS-CoV2 infection in the Japanese population: The IgA and IgG titers increased earlier than the IgM titers. Int Immunopharmacol. (2022) 103:108491. doi: 10.1016/j.intimp.2021.108491 34954559 PMC8687758

[114] Sterlin D Mathian A Miyara M Mohr A Anna F Claër L. IgA dominates the early neutralizing antibody response to SARS-CoV-2. Sci Transl Med. (2021) 13:eabd2223. doi: 10.1126/scitranslmed.abd2223 33288662 PMC7857408

[115] Boehm MK Woof JM Kerr MA Perkins SJ. The Fab and Fc fragments of IgA1 exhibit a different arrangement from that in IgG: a study by X-ray and neutron solution scattering and homology modelling. J Mol Biol. (1999) 286:1421–47. doi: 10.1006/jmbi.1998.2556 10064707

[116] Takamatsu Y Omata K Shimizu Y Kinoshita-Iwamoto N Terada M Suzuki T. SARS-CoV-2-Neutralizing Humoral IgA Response Occurs Earlier but Is Modest and Diminishes Faster than IgG Response. Microbiol Spectr. (2022) 10:e0271622. doi: 10.1128/spectrum.02716-22 36219096 PMC9769934

[117] Hennings V Thörn K Albinsson S Lingblom C Andersson K Andersson C. The presence of serum anti-SARS-CoV-2 IgA appears to protect primary health care workers from COVID-19. Eur J Immunol. (2022) 52:800–9. doi: 10.1002/eji.202149655 PMC9087394 35128644

[118] Regev-Yochay G Gonen T Gilboa M Mandelboim M Indenbaum V Amit S. Efficacy of a Fourth Dose of Covid-19 mRNA Vaccine against Omicron. N Engl J Med. (2022) 2022:02.15.22270948. doi: 10.1056/NEJMc2202542 PMC9006792 35297591

[119] GeurtsvanKessel CH Geers D Schmitz KS Mykytyn AZ Lamers MM Bogers S. Divergent SARS-CoV-2 Omicron–reactive T and B cell responses in COVID-19 vaccine recipients. Sci Immunol. (2024) 7:eabo2202. doi: 10.1126/sciimmunol.abo2202 PMC8939771 35113647

[120] Li X. Omicron: Call for updated vaccines. J Med Virol. (2022) 94:1261–3. doi: 10.1002/jmv.27530 34927258

[121] Topol EJ Iwasaki A. Operation Nasal Vaccine—Lightning speed to counter COVID-19. Sci Immunol. (2022) 7:eadd9947. doi: 10.1126/sciimmunol.add9947 35862488

[122] Mouro V Fischer A. Dealing with a mucosal viral pandemic: lessons from COVID-19 vaccines. Mucosal Immunol. (2022) 15:584–94. doi: 10.1038/s41385-022-00517-8 PMC9062288 35505121

[123] Afkhami S D’Agostino MR Zhang A Stacey HD Marzok A Kang A. Respiratory mucosal delivery of next-generation COVID-19 vaccine provides robust protection against both ancestral and variant strains of SARS-CoV-2. Cell. (2022) 185:896–915.e19. doi: 10.1016/j.cell.2022.02.005 35180381 PMC8825346

[124] Tang J Zeng C Cox TM Li C Son YM Cheon IS. Respiratory mucosal immunity against SARS-CoV-2 following mRNA vaccination. Sci Immunol. (2022) 7:eadd4853. doi: 10.1126/sciimmunol.add4853 35857583 PMC9348751

[125] Bleier BS Ramanathan MJ Lane AP. COVID-19 vaccines may not prevent nasal SARS-coV-2 infection and asymptomatic transmission. Otolaryngol Neck Surg Off J Am Acad Otolaryngol Neck Surg. (2021) 164:305–7. doi: 10.1177/0194599820982633 33320052

[126] Madhavan M Ritchie AJ Aboagye J Jenkin D Provstgaad-Morys S Tarbet I. Tolerability and immunogenicity of an intranasally-administered adenovirus-vectored COVID-19 vaccine: An open-label partially-randomised ascending dose phase I trial. eBioMedicine. (2022) 85:104298. doi: 10.1016/j.ebiom.2022.104298 36229342 PMC9550199

[127] Altimmune Inc. Altimmune announces update on adCOVID^TM^ phase 1 clinical trial. Pharmathene, Inc. Historical Press Releases. (2021). Available online at: https://ir.altimmune.com/news-releases/news-release-details/altimmune-announces-update-adcovidtm-phase-1-clinical-trial.

[128] McCallum M Walls AC Sprouse KR Bowen JE Rosen LE Dang HV. Molecular basis of immune evasion by the Delta and Kappa SARS-CoV-2 variants. Sci (80-. ). (2021) 374:1621–6. doi: 10.1126/science.abl8506 PMC12240541 34751595

[129] Garcia-Beltran WF St. Denis KJ Hoelzemer A Lam EC Nitido AD Sheehan ML. mRNA-based COVID-19 vaccine boosters induce neutralizing immunity against SARS-CoV-2 Omicron variant. Cell. (2022) 185:457–66.e4. doi: 10.1016/j.cell.2021.12.033 PMC8733787 34995482

[130] Wilhelm A Widera M Grikscheit K Toptan T Schenk B Pallas C. Limited neutralisation of the SARS-CoV-2 Omicron subvariants BA.1 and BA.2 by convalescent and vaccine serum and monoclonal antibodies. eBioMedicine. (2022) 82:104158. doi: 10.1016/j.ebiom.2022.104158 35834885 PMC9271884

[131] Mannar D Saville JW Zhu X Srivastava SS Berezuk AM Tuttle KS. SARS-CoV-2 Omicron variant: Antibody evasion and cryo-EM structure of spike protein–ACE2 complex. Sci (80-. ). (2022) 375:760–4. doi: 10.1126/science.abn7760 PMC9799367 35050643

[132] Carreño JM Alshammary H Tcheou J Singh G Raskin AJ Kawabata H. Activity of convalescent and vaccine serum against SARS-CoV-2 Omicron. Nature. (2022) 602:682–8. doi: 10.1038/s41586-022-04399-5 35016197

[133] Planas D Saunders N Maes P Guivel-Benhassine F Planchais C Buchrieser J. Considerable escape of SARS-CoV-2 Omicron to antibody neutralization. Nature. (2022) 602:671–5. doi: 10.1038/s41586-021-04389-z 35016199

[134] Hoffmann M Krüger N Schulz S Cossmann A Rocha C Kempf A. The Omicron variant is highly resistant against antibody-mediated neutralization: Implications for control of the COVID-19 pandemic. Cell. (2022) 185:447–456.e11. doi: 10.1016/j.cell.2021.12.032 35026151 PMC8702401

[135] Cele S Jackson L Khoury DS Khan K Moyo-Gwete T Tegally H. Omicron extensively but incompletely escapes Pfizer BNT162b2 neutralization. Nature. (2022) 602:654–6. doi: 10.1038/s41586-021-04387-1 PMC8866126 35016196

[136] Liu L Iketani S Guo Y Chan JF-W Wang M Liu L. Striking antibody evasion manifested by the Omicron variant of SARS-CoV-2. Nature. (2022) 602:676–81. doi: 10.1038/s41586-021-04388-0 35016198

[137] VanBlargan LA Errico JM Halfmann PJ Zost SJ Crowe JE Purcell LA. An infectious SARS-CoV-2 B.1.1.529 Omicron virus escapes neutralization by therapeutic monoclonal antibodies. Nat Med. (2022) 28:490–5. doi: 10.1038/s41591-021-01678-y PMC8767531 35046573

[138] Evans JP Zeng C Qu P Faraone J Zheng Y-M Carlin C. Neutralization of SARS-coV-2 omicron sub-lineages BA.1, BA.1.1, and BA.2. Cell Host Microbe. (2022) 30:1093–102.e3. doi: 10.1016/j.chom.2022.04.014 PMC9035359 35526534

[139] Bruel T Hadjadj J Maes P Planas D Seve A Staropoli I. Serum neutralization of SARS-CoV-2 Omicron sublineages BA.1 and BA.2 in patients receiving monoclonal antibodies. Nat Med. (2022) 28:1297–302. doi: 10.1038/s41591-022-01792-5 35322239

[140] Mykytyn AZ Rissmann M Kok A Rosu ME Schipper D Breugem TI. Antigenic cartography of SARS-CoV-2 reveals that Omicron BA.1 and BA.2 are antigenically distinct. Sci Immunol. (2022) 7:02.23.481644. doi: 10.1126/sciimmunol.abq4450 PMC9273038 35737747

[141] Zhou H Dcosta BM Landau NR Tada T. Resistance of SARS-coV-2 omicron BA.1 and BA.2 variants to vaccine-elicited sera and therapeutic monoclonal antibodies. Viruses. (2022) 14:1334. doi: 10.3390/v14061334 35746806 PMC9228817

[142] Iketani S Liu L Guo Y Liu L Chan JF-W Huang Y. Antibody evasion properties of SARS-CoV-2 Omicron sublineages. Nature. (2022) 604:553–6. doi: 10.1038/s41586-022-04594-4 PMC9021018 35240676

[143] Yu J Collier AY Rowe M Mardas F Ventura JD Wan H. Neutralization of the SARS-coV-2 omicron BA.1 and BA.2 variants. N Engl J Med. (2022) 386:1579–80. doi: 10.1056/NEJMc2201849 PMC9006770 35294809

[144] The Lancet Infectious Diseases. Why hybrid immunity is so triggering. Lancet Infect Dis. (2022) 22:1649. doi: 10.1016/S1473-3099(22)00746-0 36372089 PMC9648977

[145] Stein C Nassereldine H Sorensen RJD Amlag JO Bisignano C Byrne S. Past SARS-CoV-2 infection protection against re-infection: a systematic review and meta-analysis. Lancet. (2023) 401:833–42. doi: 10.1016/S0140-6736(22)02465-5 PMC9998097 36930674

[146] Kang S Brown HM Hwang S. Direct antiviral mechanisms of interferon-gamma. Immune Netw. (2018) 18:e33. doi: 10.4110/in.2018.18.e33 30402328 PMC6215902

[147] Redd AD Nardin A Kared H Bloch EM Abel B Pekosz A. Minimal crossover between mutations associated with omicron variant of SARS-coV-2 and CD8 + T-cell epitopes identified in COVID-19 convalescent individuals. MBio. (2022) 13:e0361721. doi: 10.1128/mbio.03617-21 35229637 PMC8941890

[148] Liu J Chandrashekar A Sellers D Barrett J Jacob-Dolan C Lifton M. Vaccines elicit highly conserved cellular immunity to SARS-CoV-2 Omicron. Nature. (2022) 603:493–6. doi: 10.1038/s41586-022-04465-y PMC8930761 35102312

[149] Madden DR. The three-dimensional structure of peptide-MHC complexes. Annu Rev Immunol. (1995) 13:587–622. doi: 10.1146/annurev.iy.13.040195.003103 7612235

[150] Allen JD Chawla H Samsudin F Zuzic L Shivgan AT Watanabe Y. Site-specific steric control of SARS-coV-2 spike glycosylation. Biochemistry. (2021) 60:2153–69. doi: 10.1021/acs.biochem.1c00279 PMC8262170 34213308

[151] Larsen F Madsen HO Sim RB Koch C Garred P. Disease-associated mutations in human mannose-binding lectin compromise oligomerization and activity of the final protein. J Biol Chem. (2004) 279:21302–11. doi: 10.1074/jbc.M400520200 14764589

[152] Garred P Larsen F Madsen HO Koch C. Mannose-binding lectin deficiency—revisited. Mol Immunol. (2003) 40:73–84. doi: 10.1016/S0161-5890(03)00104-4 12914814

[153] Garred P Mollnes TE Lea T Fischer E. Characterization of a monoclonal antibody MoAb bH6 reacting with a neoepitope of human C3 expressed on C3b, iC3b, and C3c. Scand J Immunol. (1988) 27:319–27. doi: 10.1111/j.1365-3083.1988.tb02353.x 2451273

[154] Mollnes TE Lea T Harboe M Tschopp J. Monoclonal antibodies recognizing a neoantigen of poly(C9) detect the human terminal complement complex in tissue and plasma. Scand J Immunol. (1985) 22:183–95. doi: 10.1111/j.1365-3083.1985.tb01870.x 4035298

[155] Hansen CB Jarlhelt I Hasselbalch RB Hamm SR Fogh K Pries-Heje MM. Antibody-dependent neutralizing capacity of the SARS-CoV-2 vaccine BNT162b2 with and without previous COVID-19 priming. J Internal Med. (2021) 290:1272–4. doi: 10.1111/joim.v290.6 PMC8447364 34237190

[156] Pérez-Alós L Hansen CB Almagro Armenteros JJ Madsen JR Heftdal LD Hasselbalch RB. Previous immunity shapes immune responses to SARS-CoV-2 booster vaccination and Omicron breakthrough infection risk. Nat Commun. (2023) 14:5624. doi: 10.1038/s41467-023-41342-2 37699890 PMC10497567

[157] Hadfield J Megill C Bell SM Huddleston J Potter B Callender C. Nextstrain: real-time tracking of pathogen evolution. Bioinformatics. (2018) 34:4121–3. doi: 10.1093/bioinformatics/bty407 PMC6247931 29790939

[158] Sagulenko P Puller V Neher RA. TreeTime: Maximum-likelihood phylodynamic analysis. Virus Evol. (2018) 4:vex042. doi: 10.1093/ve/vex042 29340210 PMC5758920

[159] Walls AC Park Y-JJ Tortorici MA Wall A McGuire AT Veesler D. Structure, function, and antigenicity of the SARS-coV-2 spike glycoprotein. Cell. (2020) 181:281–292.e6. doi: 10.1016/j.cell.2020.02.058 32155444 PMC7102599

[160] Berman HM Westbrook J Feng Z Gilliland G Bhat TN Weissig H. The protein data bank. Nucleic Acids Res. (2000) 28:235–42. doi: 10.1093/nar/28.1.235 PMC102472 10592235

[161] Sehnal D Bittrich S Deshpande M Svobodová R Berka K Bazgier V. Mol* Viewer: modern web app for 3D visualization and analysis of large biomolecular structures. Nucleic Acids Res. (2021) 49:W431–7. doi: 10.1093/nar/gkab314 PMC8262734 33956157

