# Supplementary material for: Unraveling the impact of SARS-CoV-2 mutations on immunity: insights from innate immune recognition to antibody and T cell responses
Source: Front Immunol. 2024 Dec 10;15:1412873. doi: 10.3389/fimmu.2024.1412873 (PMC11666439; doi:10.3389/fimmu.2024.1412873)
Supplement: Supplementary file 1 [file DataSheet1.docx]

**Supplementary Materials**


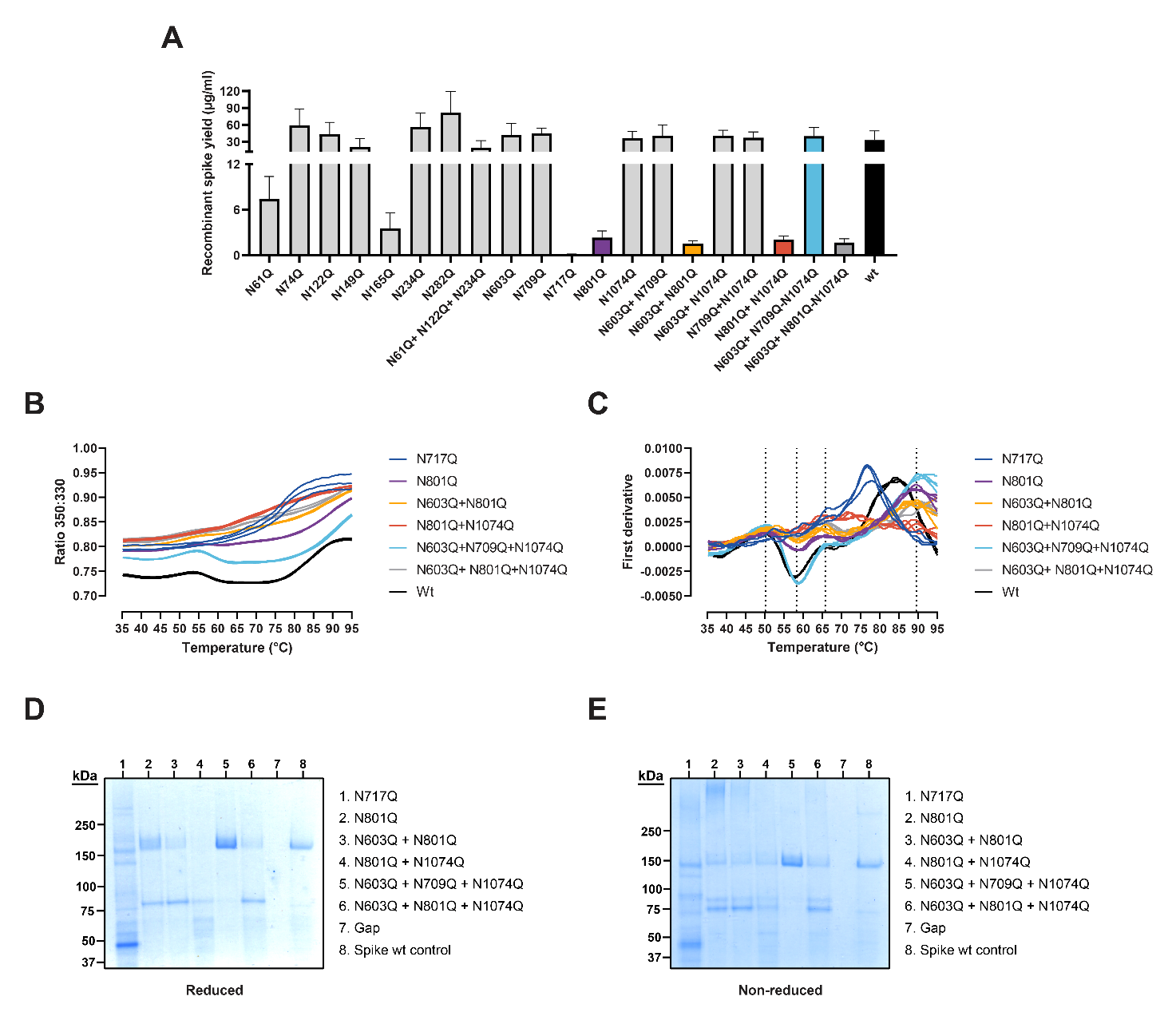


**Supplementary figure 1. Impact of removal of the glycans in the 717 and 801 position. (A)** Recombinant spike protein yield in ExpiCHO cell culture supernatants determined by ELISA using coated ACE-2 (1 µg/ml) for capture, and the anti-spike mAb clone 53 (2 µg/ml) followed polyclonal rabbit anti-mouse-HRP conjugate for detection. **(B-C)** Thermal denaturation profiles of IMAC-purified spike glycan variants. Data are represented as individual 350:330 nm ratio curves (B) and the first derivative of the ratio (C) from three repeats. Vertical dotted lines represent the average Ti of the control proteins without the N717Q or N801Q mutations (i.e. N603Q + N709Q + N1074Q and the wt). **(D-E)** Coomassie stain of IMAC-purified spike glycan variants separated by SDS-PAGE under reducing (D) and non-reducing (E) conditions.

**
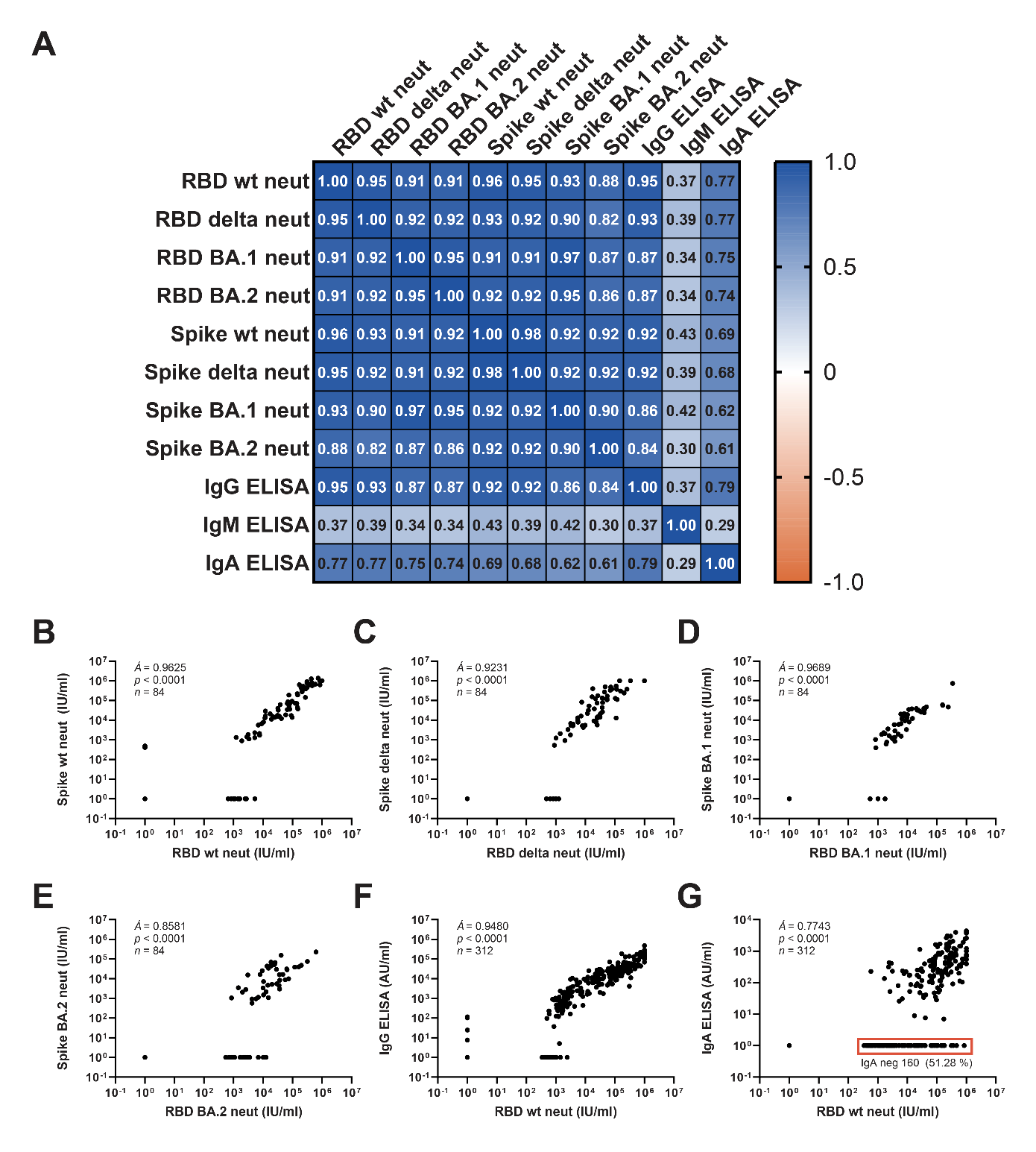
**

**Supplementary figure 2.** **Correlation analyses of nAb responses against RBD and spike variants and RBD-specific total antibody titers (IgG, IgM, IgA).** (**A)** Spearman correlation matrix. **(B–E)** Spearman correlation between RBD and spike wt (B), Delta (C), BA.1 (D), and BA.2 (E). **(F)** Spearman correlation between RBD wt neutralization and anti-RBD IgG titers. **(G)** Spearman correlation between RBD wt neutralization and anti-RBD IgA titers. Red box highlights IgA samples below the lower limit of quantification. Neut: neutralization.

**
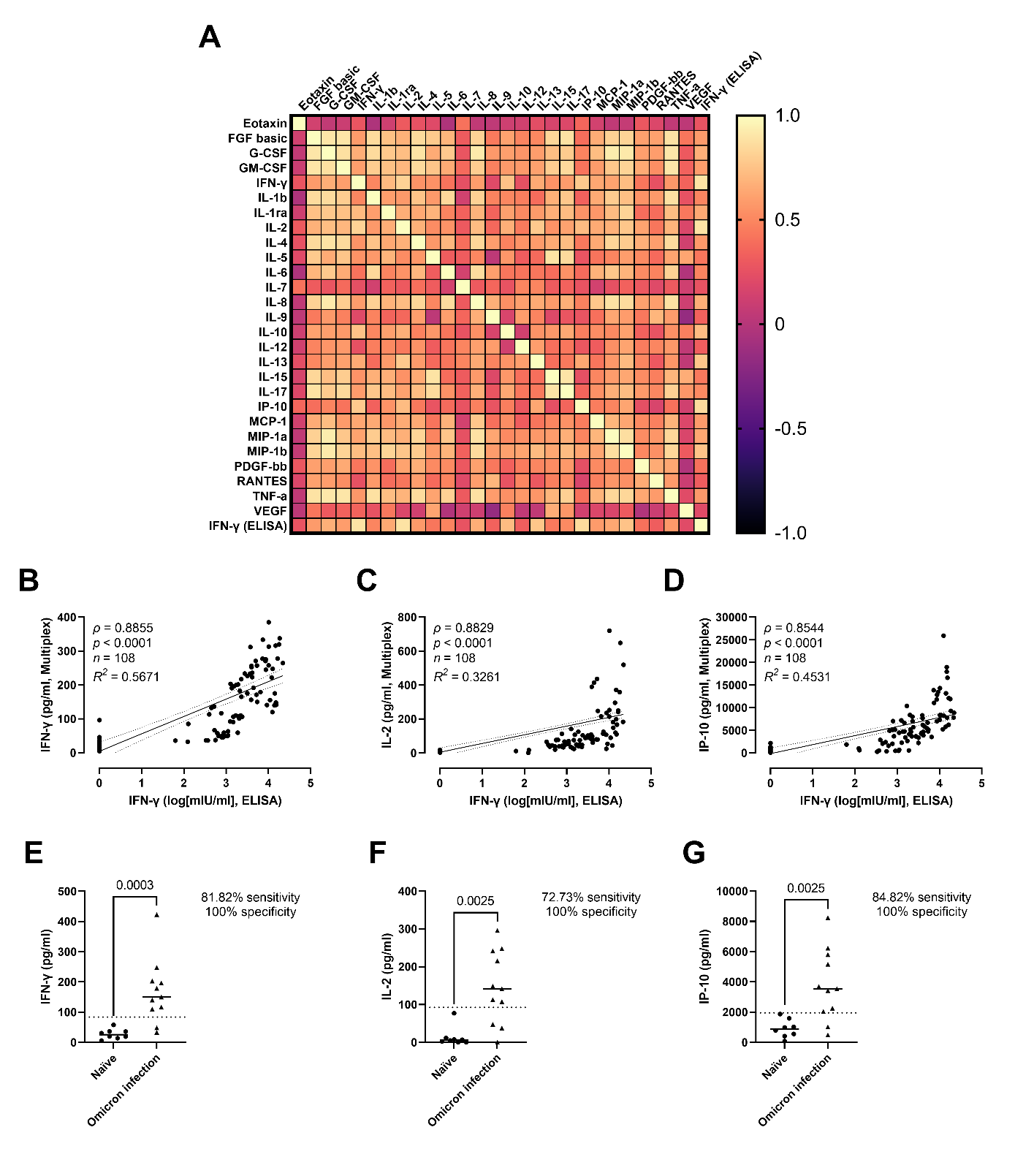
**

**Supplementary figure 3.** **Cytokine profiling in whole blood after T cell peptide MP stimulation. (A)** Spearman correlation matrix of secreted cytokines measured by Luminex and ELISA. Color gradient represents the Spearman’s correlation coefficient (ρ). **(B–D)** Correlation and linear regression analyses of IFN- γ levels, measured by ELISA, and Luminex measurements of IFN- γ (B), IL-2 (C), and IP-10 (D). Solid lines represent the linear fit. Dotted lines represent the 95 % CI. **(E–G)** Mann-Whitney tests of IFN-γ, IL-2, and IP-10 release after stimulation with CD4RE peptide pool in infection-naïve (*n* = 8) or in individuals with a recent Omicron infection (*n* = 11). Dotted line represents the threshold for positivity determined by ROC curve analyses.
